# Synthetic Biology‐Based Engineering Cells for Drug Delivery

**DOI:** 10.1002/EXP.20240095

**Published:** 2025-01-16

**Authors:** Wenzhe Yi, Shuangshuang Hu, Xindi Qian, Wenlu Yan, Yaping Li

**Affiliations:** ^1^ State Key Laboratory of Drug Research and Center of Pharmaceutics Shanghai Institute of Materia Medica Chinese Academy of Sciences Shanghai China; ^2^ University of Chinese Academy of Sciences Beijing China; ^3^ School of Life Sciences Jilin University Changchun China; ^4^ Shandong Laboratory of Yantai Drug Discovery Bohai Rim Advanced Research Institute for Drug Discovery Yantai China

**Keywords:** drug delivery, engineered cells, synthetic biology

## Abstract

Although drug delivery technology has promoted the clinical translation of small molecule drugs, there is an urgent need for advanced delivery systems to overcome complex physiological barriers and the increasing development of biological drugs. This review overviews the emerging applications of synthetic biology‐based engineered cells for drug delivery. We first introduce synthetic biology strategies to engineer cells for biological drug delivery and discuss the benefits in terms of specificity, intelligence, and controllability. Furthermore, we highlight the cutting‐edge advancements at the convergence of synthetic biology and nanotechnology in drug delivery. Nanotechnology expands the engineering design and construction concepts of synthetic biology, and synthetic biology drives the development for biotechnology‐driven nanomaterial synthesis. In the future, synthetic biology‐based engineered cells may be developed to be more modular, standardized, and intelligent, leading to significant breakthroughs in the construction of advanced drug delivery systems.

## Graphical abstract

Synthetic biology‐based therapeutic cells for drug delivery: Engineered cells effectively transport and release drugs through their intelligence and controllability, such as feedback pathways and external stimuli (e.g., light and ultrasound). The integration of nanotechnology, including nanoparticles, biofilms, and artificial cells, enhances specificity and versatility. Together, synthetic biology and nanotechnology are paving the way for advanced intelligent therapeutic systems.

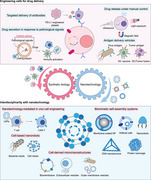

## 1 Introduction

Drug delivery technology has significantly improved the efficacy and the clinical application of drugs [1, 2]. Nanomedicine has revolutionized the targeted delivery of small‐molecule drugs, but it faces obstacles due to the biological barriers and complex nano‐bio interactions in the body [3, 4, 5]. In addition, the development of nanocarriers for biological drugs (such as nucleic acids, vaccines, recombinant proteins, and antibodies) is still in its early stages and faces several challenges. Therefore, alternative drug delivery technologies need to be developed to meet the advancing requirements of drug delivery.

Synthetic biology integrates molecular biology and cutting‐edge engineering principles to design, engineer, or even recreate living organisms with capabilities beyond those found in nature [6, 7]. In recent years, synthetic biology‐based cells have gradually emerged as a new branch of medicine alongside chemical and biological drugs [8, 9, 10, 11]. For example, the emergence of the first FDA‐approved chimeric antigen receptor (CAR‐T) therapy, Tisagenlecleucel (Kymriah), in 2017 marked a significant milestone in cell therapy driven by synthetic biology [12]. These cells have unique advantages in long circulation, targeting, biocompatibility, and overcoming physiological barriers in the body [13]. Synthetic biology programming further gives them the ability to synthesize and release biological drugs in response to pathological signals. Therefore, synthetic biology‐based cells can serve as delivery systems for biological drugs, establishing the groundwork for the creation of an “intelligent” cell‐based therapy platform.

With the rapid advancement of engineering concepts in drug delivery technology, the inevitable outcome is the convergence of nanotechnology and synthetic biology. An advanced understanding of nanotechnology for biomedical purposes, combined with deliberate design using synthetic biology, offers a completely new approach to drug delivery. On one hand, synthetic biology‐based cells can generate unique biological functional nanomaterials, driving the development of new theories for biotechnology‐driven nanomaterial synthesis. On the other hand, using nanomaterials to enhance cell function or simulate live cells expands the engineering design and construction concepts of synthetic biology. Till now, T cells (NCT03815682) and red blood cells (RBC) (NCT04372706) loaded with nanoparticles have shown significant progress in clinical research, demonstrating great therapeutic potential.

In this review, we overview the current progress of synthetic biology‐based engineered cells for drug delivery. The emerging synthetic biology strategies to engineer cells for biological drug delivery are categorized and summarized. Furthermore, we highlight the cutting‐edge progress in the cross‐disciplinary field of synthetic biology and nanotechnology in drug delivery, including nanotechnology‐mediated in vivo cell engineering, cell‐based nanorobots, cell‐derived micro/nanostructures, and biomimetic self‐assembly systems. In the end, we look forward to technological challenges and necessary advancements of the convergence of synthetic biology and nanotechnology, which may provide insights into the rational design of engineering cells in drug delivery.

## 2 Core Concepts of Synthetic Biology

### 2.1 The Top‐Down Approach: Cell Engineering

**FIGURE 1:**
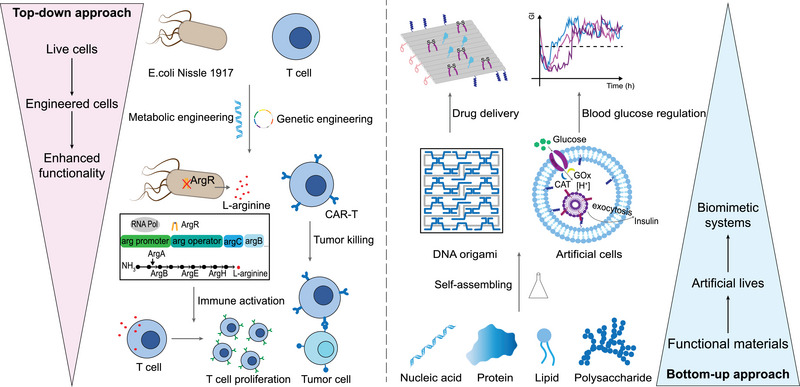
Schematic illustration of the concept of synthetic biology. The top‐down approach (left) focuses on modifying living cells, typically by introducing artificial components through genetic or metabolic engineering, to enhance or impart new functions to the cells based on the researchers' intentions. The bottom‐up approach (right) involves assembling isolated biological molecules and synthetic modules to construct artificial biomimetic systems that mimic the functions of natural living organisms for drug delivery and disease treatment. *N*‐acetylglutamate kinase (ArgB); *N*‐acetyl‐γ‐glutamino‐phosphate reductase (ArgC); L‐ornithine acetyltransferase (ArgE); Arginine succinate lyase (ArgH).

The progress in genetic and metabolic engineering has greatly advanced the top‐down modification of living cells, such as controlling gene expression to give cells the desired functions (disease targeting, environmental response, cytokine secretion, etc.) [14] (Figure 1). The synthetic biological systems obtained by remodeling living cells can directly serve as a means of disease treatment and provide possibilities for the synthesis and targeted delivery of protein drugs (such as antibodies and cytokines) [15]. We will briefly introduce the top‐down approach with a primary emphasis on the significant advancements in genetic and metabolic engineering. Gene editing technologies alter the traits of organisms through precise manipulation of DNA sequences. Zinc‐finger nucleases (ZFNs) [16], transcription activator‐like (TAL) effector nucleases (TALENs) [17], and clustered regularly interspaced short palindromic repeat‐associated 9 nucleases (CRISPR‐Cas9) [18] are currently being developed for the construction and modulation of synthetic gene circuits to encode the functions of living cells. The CRISPR‐Cas system is the most powerful gene editing platform and was first used as a genome editing tool in mammalian cells in 2013 [19]. Since then, the CRISPR‐Cas9 system has been widely used to remodel living cells for disease treatment [20, 21]. For example, researchers have used CRISPR‐Cas9 genome engineering to create a self‐regulating synthetic gene circuit in induced pluripotent stem cells (iPSCs) [22]. This circuit controls the release of anti‐cytokine biologics by sensing changes in endogenous inflammatory cytokine levels, triggering the corresponding therapeutic response. Furthermore, a phase I clinical trial demonstrated the safety and excellent effect of CRISPR‐Cas9‐based programmed cell death 1 (PD‐1) knockout T cells in a group of advanced non‐small‐cell lung cancer patients (NCT02793856), highlighting the clinical translational potential of genetically engineered therapeutic cells [23].

Metabolic engineering provides a powerful tool for using recombinant DNA technology to modify cellular metabolic networks to efficiently produce specific natural products such as amino acids, chemical raw materials, pharmaceutical intermediates, and biomacromolecules [24]. By optimizing, introducing, or knocking out key enzymes or genes in the pathway of product synthesis, therapeutic cells can specifically respond to the external environment, achieve the synthesis and release of target metabolic products, and expand the treatment opportunities for difficult diseases such as malignant tumors. L‐arginine is a key determinant for effective anti‐tumor T‐cell response [25, 26]. However, the low concentration of L‐arginine within the tumor restricts T cell activity [27]. To address this issue, a probiotic strain of *Escherichia coli Nissle 1917* (ECN) has been engineered to delete the arginine repressor gene (ArgR) and integrate a mutation in *N*‐acetylglutamate synthase (ArgA) that is not subject to negative feedback regulation [28]. It colonizes in the tumor and continuously converts accumulated metabolic waste, ammonia, into L‐arginine, increasing the infiltration of T cells in the tumor. Furthermore, by designing a toggle switch to dynamically redirect cellular metabolism towards L‐arginine biosynthesis from the tricarboxylic acid cycle, significant improvements can be made in the production efficiency of L‐arginine in *Escherichia coli* for industrial purposes [29]. Recently, Dermatobacterium acnes, the most abundant commensal in the skin, has been modified to secrete neutrophil gelatinase‐associated lipid‐transporting proteins to regulate skin sebum production [30]. This suggests a more secure and effective approach by engineering the cells from the host source, minimizing the risk of immune rejection and adverse reactions. In conclusion, metabolic engineering‐based therapeutic cells have provided new vitality into biomedical engineering, with great potential for application.

### 2.2 The Bottom‐Up Approach: Artificial Biological System

The bottom‐up strategy emphasizes the use of non‐natural biological components to create new biological systems in vitro [31]. From an engineering perspective, multiple independent modules are assembled according to a certain strategy to obtain entirely new complex systems. Materials such as nucleic acids, proteins, and organic polymers can be assembled into nano‐reactors or compartments, further used for disease treatment, drug delivery, and even the construction of artificial cells [32, 33] (Figure 1).

DNA is not only the genetic material of life, but also serves as a self‐assembling material, creating unique structures based on highly precise base pairing principles [34]. Traditional DNA nanocages and DNA origami techniques endow nucleic acids with high editability, making them suitable as vaccine components or drug delivery platforms [35]. For example, folding DNA into structures resembling those of the human immunodeficiency virus (HIV), with surfaces covered in HIV proteins or antigens, can elicit strong immune responses [36]. Surface lipid modification of DNA origami structures encoding the tumor suppressor gene p53 allows for efficient cellular uptake and transcription, leading to significant upregulation of active p53 protein expression and inhibition of tumor growth [37]. Similarly, proteins can be assembled into protein nanoparticles or virus‐like particles for use in disease treatment and drug delivery [38, 39].

Artificial cells are built from scratch where organic or inorganic materials are chemically assembled to construct cell‐like structures with certain simulated cellular functions [40]. In 2010, Craig Venter and others created the simplest synthetic cell, “Synthia,” for the first time, and subsequently achieved the normal division and proliferation of the cells [41, 42, 43]. This laid the foundation for understanding how life works. In addition, nanovesicles assembled using lipids and polymers can also be used to assemble artificial cells that mimic the functions of natural cells [44]. For example, artificial beta cells based on lipid vesicles can mimic the function of pancreatic islet cells. The assembled glucose‐related metabolic system and pH responsiveness are utilized to mimic insulin release and effectively respond to hyperglycemia [45]. Exosome‐thermosensitive liposome hybrid artificial cells can encapsulate granulocyte‐macrophage colony‐stimulating factor (GM‐CSF) and docetaxel to tumor sites [46]. In summary, the construction of a range of functional modules has created numerous opportunities for simulating complex living systems, as well as providing a rich arsenal of tools for disease treatment and drug delivery.

## 3 Synthetic Biology Strategies to Engineer Cells for Biological Drug Delivery

Biological drugs mainly consist of nucleic acids, vaccines, recombinant proteins, and antibodies. Due to their high specificity, biological drugs have significant advantages in treating serious diseases. However, their complex isolation and purification, strong immunogenicity, intricate crystal structure, and challenges in crossing biological barriers have limited their application [47, 48, 49]. Synthetic biology‐based engineered cells serve as natural processing platforms, enabling the synthesis of biological drugs. Driven by the inherent chemotactic properties, engineered cells can overcome multiple biological barriers and deliver drugs to the site of the disease. In this section, we highlight the synthetic biology strategies for the delivery of biological drugs based on engineered cells (Table 1).

**TABLE 1:** Representative examples of synthetic biology strategies to engineer cells for biological drug delivery.

Synthetic biology strategies	Engineered methods	Types of cells	Cell modification	Efficiencies	Refs.
Targeted delivery of antibodies	Genetic engineering	Platelets	Overexpression of PD‐1	Targeted delivery of PD‐1 thus enhancing the tumor‐killing ability of T cells	[50]
	Genetic engineering	MK progenitor cells	Overexpression of PD‐L1	Inhibit autoreactivity T cells thus reversing type 1 diabetes mellitus	[51]
	Genetic engineering	Probiotics	Synthesis of PD‐L1 and CTLA‐4 blocking nanoparticles	Continuous delivery of immune checkpoint inhibitors locally to the tumor	[52]
	Genetic engineering	*E. coli Nissle 1917*	Lyse and release CD47nb	Increase the viability of tumor‐infiltrating T‐cells and promote rapid tumor regression	[53]
Drug secretion in response to pathological signals	Genetic engineering	HEK293 cells	Synthesis of insulin	Respond to high levels of glucose and maintain glycemic homeostasis	[54]
	Metabolic engineering	*E. coli Nissle 1917*	Delete the ArgR	Convert ammonia into L‐arginine thus increasing the infiltration of T cells	[28]
	Genetic engineering	T cells	synNotch‐CAR ‐T	Locally induced IL‐2 production effectively enhanced the infiltration of CAR‐T cells and cleared immune‐rejecting tumors	[55]
	Genetic engineering	*E. coli Nissle 1917*	Overexpression of catalase and superoxide dismutase	Effectively clear ROS for treating intestinal inflammation	[56]
Drug release under manual control	Genetic engineering	*E. coli Nissle 1917*	Contain promoters that respond to blue light	Achieve the photo‐controlled synthesis of TGF‐β1 and IFN‐γ	[57]
	Genetic engineering	*E. coli MG1655*	Contain ultrasound responsive and IFN‐γ temperature‐controlled gene expression circuit	Achieve the ultrasound‐controlled synthesis of IFN‐γ	[58]
	Genetic engineering nanoparticle binding	*E. coli BL21*	Contains a heat‐sensitive promoter, Fe_3_O_4_@lipid	Magnetic drive enrichment to tumor site, the magnetic signal is converted to heat, which releases the anti‐CD47 nanoantibodies	[59]
	Genetic engineering	β cells	Electroβ cells	Release of insulin after electrical stimulation and restore normal blood sugar levels in type 1 diabetic mice	[60]
As antigen delivery vehicles	Genetic engineering	Lactobacillus	Overexpression of Flt3L and OX40L	Releases of tumor‐specific antigens after killing cancer cells, and promotes antigen presentation to DCs	[61]
	Genetic engineering	*E. coli*	Contain a promoter induced by the monosaccharide arabinose, Ag and mFc	The OMVs can be taken up by antigen‐presenting cells in the lamina propria, activating a strong anti‐tumor immune response	[62]
	Genetic engineering	DC	DC/tumor fusion cells (FCs)	PD‐1 Nb20 in synergy with the DC/tumor‐FC vaccine improves the antitumor activity of T‐cells against multiple tumor types	[63]
	Genetic engineering	Tumor cells	Reprogram to TR‐APC	Process and present endogenous TAAs	[64]

## 4 Targeted Delivery of Antibodies

Programmed cell death ligand 1 (PD‐L1) is widely present in tumor cells and some immune cells (e.g. dendritic cells), and it is able to bind to PD‐1 of T lymphocytes, thus inhibiting the immune recognition function of T lymphocytes [65]. Although immune checkpoint blockade (ICB) has been used in cancer therapy, the clinical response rate to ICB therapy is still low due to its fragile structure and poor tumor targeting [66, 67].

**FIGURE 2:**
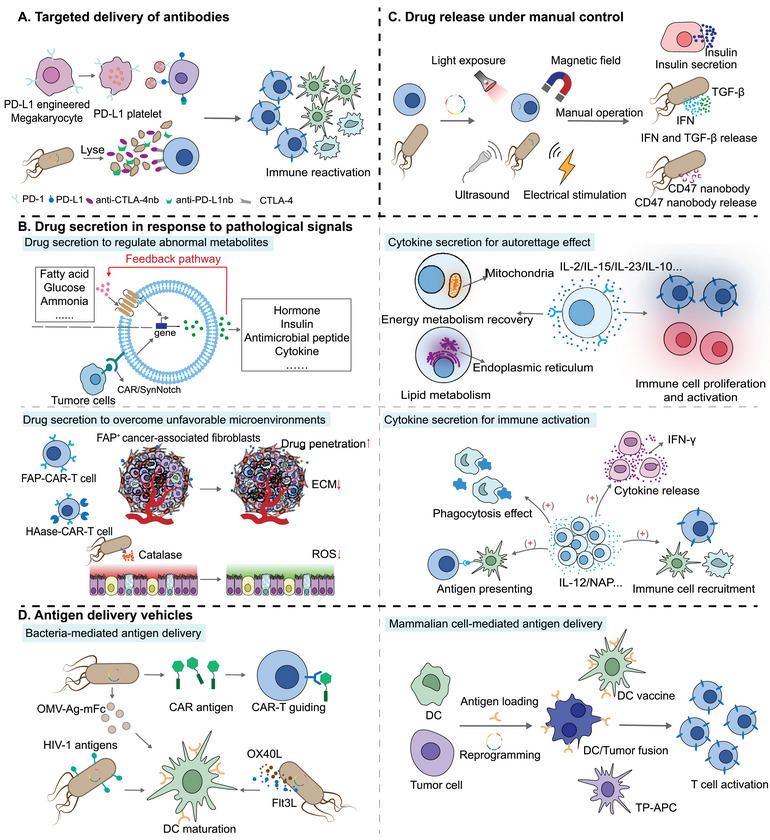
Synthetic biology strategies to engineer cells for biological drug delivery. (A) Targeted delivery of antibodies. (B) Drug secretion in response to pathological signals. Synthetic biology‐based engineered cells can secrete drugs to regulate abnormal metabolites. Moreover, engineered cells can secrete cytokines to mobilize and activate immune cells or enhance their own functions. Synthetic biology‐based cells can also secrete drugs to overcome unfavorable microenvironments. (C) Drug release under manual control. By designing specific genetic circuits, cells can be given artificial controllability, allowing for the release of drugs. (D) Performing as antigen delivery vehicles. Bacteria (left) and mammalian cells (right) can be engineered to produce tumor or pathogen antigens, triggering both innate and adaptive immune responses. Extracellular matrix (ECM).

Considering the dynamic role of platelets in tumor progression [68], the researchers attached the PD‐L1 antibody to the surface of platelets, using the tumor‐targeting ability of platelets and platelet‐activated derived particles to achieve tumor binding of the PD‐L1 antibody thus enhancing the tumor‐killing ability of T cells [69] (Figure 2A). On this basis, researchers utilized genetic engineering to generate megakaryocyte (MK) progenitor cells expressing PD‐1 or PD‐L1 to obtain PD‐1 or PD‐L1 expressing platelets for the prevention of postoperative tumor recurrence and the reversal of type 1 diabetes mellitus [50, 51]. Further research has revealed that engineered platelets can enhance the efficacy of CAR‐T cells against tumors [70]. More excitingly, CAR‐T cells secreting PD‐1 antibodies have shown preliminary therapeutic effects in the clinical treatment of advanced refractory ovarian cancer [71]. In addition, the strategy of using genetically engineered probiotics to produce nano‐antibodies targeting PD‐L1 and cytotoxic T lymphocyte‐associated antigen‐4 (CTLA‐4) to help T‐cells attack tumor cells has shown excellent results in cancers with a weak response to immunotherapy [52]. A nonpathogenic ECN strain was also genetically modified to specifically release CD47 nanoantibody antagonists (CD47 nb) in the tumor microenvironment, increasing the viability of tumor‐infiltrating T‐cells and promoting rapid tumor regression [53]. The use of engineered cells as antibody delivery vehicles not only eliminates the antibody production and purification processes but also improves the targeting and penetration of antibodies to disease lesions, thereby reducing potential off‐target effects. Although the challenges of large‐scale production of live cells need to be considered, probiotics or blood cells still show great potential for future antibody delivery.

## 5 Drug Secretion in Response to Pathological Signals

The most widespread application of synthetic biology lies in reprogramming cells to sense pathological signals, trigger response pathways, and output detectable feedbacks [72]. The use of disease‐related biomarkers to trigger the expression of therapeutic drugs is a common design strategy in synthetic biology. This allows for a gradual decrease in the concentration of pathological biomarkers. Subsequently, the feedback pathway shuts down and the concentration of drugs is gradually reduced (Figure 2B).

Endogenous metabolites in abnormal concentrations are the most commonly used pathological biomarkers [73]. High levels of metabolites stimulate engineered cells to produce therapeutic drugs that reduce metabolite levels to the normal range. For example, engineered cells can sense fatty acid levels in obese mice and produce pramlintide, an appetite suppressant hormone [74]. Engineered HEK293 cells respond to high levels of glucose and maintain glycemic homeostasis through the synthesis of insulin or low pH‐mediated insulin release [54]. An immune‐mimicking cell expressing a human Toll‐like receptor detects bacterial infection and produces antimicrobial peptides to treat methicillin‐resistant *Staphylococcus aureus* (MRSA) [75]. Engineered mesenchymal stem cells (DOPA‐MSCs) can continuously and steadily secrete dopamine, thereby restoring striatal dopamine levels for the treatment of Parkinson's disease (PD) [76]. Engineered ECN continuously converts accumulated metabolic waste, ammonia, into L‐arginine, increasing the infiltration of T cells in the tumor [28]. Recently, researchers have obtained enucleated stem cells with high expression of CXCR4, CCR2, and the selectin ligand Psgl‐1 for inflammatory lesion targeting the delivery of drugs [77]. These cells trigger signals to synthesize drugs when they enter the site of inflammation, offering the potential for alleviating inflammation. These novel engineered cells show an improved ability to sense soluble pathological signals, providing effective tools for pathological signal‐triggered drug delivery.

Cytokines are a class of biological drugs that often act as regulators of immune response and inflammation [78]. Using synthetic biology to modify cells to secrete cytokines under a disease microenvironment presents an autofrettage effect. IL‐2 or IL‐15 can significantly enhance the proliferation of CAR‐T cells in tumors thereby prolonging their survival and anti‐cancer function in vivo [79, 80]. However, the activation of bystander cells may cause toxicity. Therefore, the researchers designed circuits in which a synthetic Notch (synNotch) induces IL‐2 production to promote CAR‐T function [55]. This strictly autocrine IL‐2 CAR‐T effectively overcomes the potential side effects of IL‐2. CAR‐T cells transfected with the IL‐23b p40 gene exhibit enhanced proliferation and increased anti‐cancer activity upon activation and secretion of IL‐23 following recognition of tumor cells [81]. Compared to IL‐2 or IL‐15, CAR‐T cells that secrete IL‐23 not only demonstrate superior anti‐cancer functionality but also offer unique safety advantages due to the specific mechanism of IL‐23 production. Apart from promoting T‐cell proliferation, overcoming the T‐cell dysfunction in the tumor microenvironment (TME) is also a key focus. Recently, researchers have developed IL‐10‐secreting CAR‐T cells, which can deliver IL‐10 to TME and maintain intact mitochondrial function to increase oxidative phosphorylation in a mitochondrial succinate carrier‐dependent manner [82]. These findings not only enrich the targeted delivery vehicle toolbox of cytokine but also advance the understanding of cytokine‐regulated cell metabolism. Additionally, synthetic biology‐based cells have the ability to produce cytokines for regulating the activity of other immune cells. For example, IL‐12 expressing myeloid cells (IL‐12‐GEMy) induce the expression of genes related to the activation of adaptive immune cells and genes related to antigen processing and presentation, promoting the accumulation of activated T cells, NK cells, and DCs in the lungs to reshape the metastatic environment [83]. CAR‐T armed with neutrophil activating protein (NAP) attracts innate immune cells, induces DC cell maturation, Th1 cell polarization, and CD8^+^ T cell infiltration [84]. Building a local inflammatory microenvironment by promoting an innate immune response will facilitate the tumor‐killing function of CAR‐T. Recently, researchers have developed a dendritic cell progenitor cell (DCP) armed with IL‐12 and recombinant FMS like tyrosine kinase 3 ligand (Flt3L) (DCP‐IL‐12/Flt3L) [85]. DCP‐IL‐12/Flt3L induced interferon‐gamma (IFN‐γ)‐dependent anti‐tumor immune response and significantly upregulated intratumoral cDC1, M1‐type macrophage, and effector T‐cell ratios. Notably, DCP‐IL‐12/Flt3L has effects that go beyond those of traditional DC vaccines and are universally applicable. Since DCPs can be derived from human blood, this will aid in translating preclinical findings into potentially revolutionary cancer immunotherapies.

Tumor stromal barriers are among the key obstacles limiting the penetration of therapeutic drugs [86, 87]. Synthetic biology equips cells with weapons against immune suppressive barriers in disease environment, greatly promoting the tissue penetration of drugs. Coupling hyaluronidase to CAR‐T disrupts the tumor extracellular matrix and increases the intratumoral penetration of CAR‐T [88]. Using CAR‐T to destroy cancer‐associated fibroblasts also promotes T cells and anti‐tumor drugs into tumors [89]. Conversely, in inflammatory disease, damage to the tissue barrier and high reactive oxygen species (ROS) often lead to hyperactivation and infiltration of immune cells, exacerbating inflammatory progression. Therefore, researchers engineered ECN with overexpressed catalase and superoxide dismutase (ECN‐pE) to effectively clear ROS for treating intestinal inflammation [56]. Consequently, designing engineered cells to overcome unfavorable microenvironments holds promise for facilitating drug penetration and disease treatment.

## 6 Drug Release Under Manual Control

A challenge in the design of engineered cells is artificially manipulating cell movement and function to reduce the potential for cross‐reactivity and off‐target effects [90, 91]. Physical fields, including light, magnetic fields, electric fields, sound waves, thermal energy, and mechanical forces, provide a robust, efficient, and precise means to control cell behavior in specific times and spaces. By designing corresponding genetic circuits, cells can be endowed with artificial controllability, eliminating the need for inducers and exhibiting controllable drug release ability (Figure 2C).

Optogenetics is the use of light energy to stimulate genetic circuits in engineered cells with high precision and temporal resolution [57, 92]. ECN with promoters responding to blue light achieved the photo‐controlled synthesis of TGF‐β1 and IFN‐γ, which effectively inhibited ulcerative colitis and tumor growth in mice [57]. Ultrasound provides a platform for manipulation with high tissue permeability [93]. For example, researchers created an ultrasound‐responsive bacterium (URB) to express IFN‐γ effectively inhibit the growth of breast cancer and liver cancer [58]. Magnetogenetics is a less invasive and faster method than optogenetics [94]. Specific expression of ferritin in neurons in complex with temperature‐sensitive proteins stimulates the activity of glucose‐sensitive neurons in the hypothalamus [95]. Animals exposed to magnetic fields have elevated blood glucose concentrations and decreased insulin levels. Engineered bacteria lysed and released anti‐CD47 nanoantibodies upon stimulation with thermomagnetic signals [59]. Electrogenetics is a technology that uses electrical pulses to remotely manipulate cells. Recently, researchers have developed a method to control cells using electrical signals by expressing the L‐type voltage‐gated channel CaV1.2 and the inward rectifying potassium channel Kir2.1, coupling them to the desired output through endogenous calcium signal transduction. Using this technology, electrically sensitive human pancreatic β cells (electro β cells) were designed to release a large amount of insulin within 10 min after electrical stimulation. When implanted subcutaneously, electro β cells can restore normal blood sugar levels in type 1 diabetic mice [60]. These manipulable cells have good safety, biocompatibility, and multifunctionality, representing the future direction of synthetic biology. It also marks a significant step forward in the clinical application of controllable drug delivery and release.

## 7 Performing as Antigen Delivery Vehicles

Disease‐derived proteins, peptides, nucleic acids, or polysaccharides can be regarded as relevant antigens [96]. However, due to the poor delivery and immunogenicity of antigens, the desired immune efficacy is not achieved [97]. Fortunately, synthetic biology provides translatable strategies to transform bacteria and mammalian cells into antigen carriers for targeted delivery and precise release of antigens (Figure 2D).

For example, Lactobacillus lactis expressing the Lpp20 antigen prevented pathogenic colonization by Helicobacter pylori [98]. Lactobacillus lactis expressing the HIV Gag antigen effectively triggered mucosal humoral and cellular responses after oral immunization of mice, highlighting its potential as an anti‐HIV vaccine platform [99]. An injectable vaccine prepared by mixing inactivated virus with lectin‐loaded superparamagnetic beads and adjuvant‐loaded mesoporous silica nanoparticles is effective in a porcine model for the reduction of cutaneous abscesses caused by methicillin‐resistant *S. aureus* and for the treatment of infectious shock induced by lethal *E. coli* [100]. Not limited to anti‐infection, engineered bacteria have recently been shown to be a suitable delivery vehicle for tumor antigens, inducing potent anti‐tumor immunity. For example, direct intratumoral injection of Lactobacillus expressing a fusion protein of Flt3L and OX40 ligand (OX40L) kills cancer cells, releases large quantities of tumor‐specific antigens, and promotes antigen presentation to DCs [61]. Fusion integration of tumor antigens into bacteria allows them to secrete bacterial outer membrane vesicles (OMVs) bearing tumor antigens induced by arabinose. The OMVs can be taken up by antigen‐presenting cells in the lamina propria, activating a strong anti‐tumor immune response and greatly contributing to the development of oral tumor vaccines [62]. Similarly, cutaneous commensal bacteria armed with tumor neoantigens stimulate the production of tumor‐specific T cells and lead to complete tumor clearance with immune checkpoint therapies. Compared to exogenous bacteria, symbiotic bacteria are a safer anti‐cancer strategy because they do not trigger infections [101]. The use of probiotics to enhance CAR‐T therapy has also been explored. The researchers used synthetic biology to modify ECN so that it can infiltrate and release synthetic CAR antigens directly into the core of solid tumors [102]. CAR‐T cells then recognize the antigenic targets released by these probiotics, which in turn kill these tumor cells in situ. Allowing ECN to further release CXCL16 promotes CAR‐T infiltration and effectively inhibits the growth of multiple solid tumors. This study shows that probiotics can enhance the effectiveness of CAR‐T therapy regardless of tumor‐associated antigens, offering a significant demonstration of a potential treatment approach for diverse cold tumors.

Similarly, mammalian cells also show great potential as antigen‐delivery vectors after modification. The most common method is to present antigens to the surface of antigen‐presenting cells (APCs). DCs are capable of loading various antigens from within the body, such as whole tumor lysate and peptides [103]. Several DC subtypes have been utilized for vaccination purposes [104]. Recent phase I clinical trials have utilized cDC2s and plasmacytoid DCs, demonstrating that these methods are safe and well‐tolerated, and induce specific immunity in some patients [105, 106, 107, 108]. A new type of DC/tumor fusion cells (FCs) also shows improved antigens presentation and induces sustained T cell response [63]. Macrophage‐based therapeutic vaccines are an alternative to engineered DCs and have been tested in the clinic against multiple cancer types [109]. In addition, reprogramming tumor cells into antigen‐presenting cells is expected to activate polyclonal effector T cells, considering that tumor cells serve as the most initial and comprehensive reservoir of tumor antigens. Direct transformation of cancer cells into tumor‐reprogrammed APC (TR‐APC) using bone marrow lineage reprogramming activates endogenous tumor‐associated antigens (TAAs)‐specific T cells for leukemia therapy [64]. Direct reprogramming using the transcription factors PU.1, IRF8, and BATF3 (PIB) can transform cancer cells into immunogenic cDC1‐like cells with antigen presentation and reduced tumorigenicity [110]. Expression of PIB in cancer cells drives overall transcriptional and epigenetic remodeling and the establishment of cDC1 morphology, immunophenotype, and function, leading to tumor antigen presentation. This outstanding technology will significantly reduce the cost of immunotherapy and could be a promising treatment in the future.

## 8 Cross‐Disciplinary of Synthetic Biology and Nanotechnology in Drug Delivery

Nanotechnology has emerged as a pivotal link between materials science and biology, driving technological innovation in biomedicine [111, 112, 113]. It has facilitated advancements in gene editing, vaccine/drug delivery, in vivo imaging, and tissue engineering [114, 115, 116]. In this section, we highlight recent progress of the cross‐disciplinary of synthetic biology and nanotechnology in drug delivery (Table 2). Nanotechnology aids in the effective delivery of synthetic biological components across physiological barriers to reach target locations. Additionally, the chemical, physical, and biological properties of nanomaterials make it easier to manipulate engineered cells both in vivo and in vitro compared to genetic circuits. Correspondingly, cell‐derived nanovesicles and biomacromolecules can be autonomously designed with the help of synthetic biology, leading to significant breakthroughs in biomedical applications. Guided by synthetic biology, nanomaterial‐based self‐assembled nanocomponents mimic the characteristics of living cells, such as enzyme‐catalyzed reactions and stimulus responsiveness, offering new design perspectives for the construction of nanorobots and artificial cells.

**TABLE 2:** Representative examples of the cross‐disciplinary of synthetic biology and nanotechnology in drug delivery.

In vivo engineering					
Carriers	Delivery targets	Engineered cells	Efficiencies	Indications	Refs.
Nano‐porous silicon rod scaffolds	IL‐2 and T‐cell‐activating antibodies	T‐cells	T cells expand exponentially and remain highly active	Xenograft lymphoma	[117]
Multifunctional alginate scaffold	Viral particles encoding the CAR	T‐cells	Expedite the reprogramming of T cells and reduce the preparation time for CAR‐T cells	Xenograft lymphoma	[118]
Injectable hydrogel	pCAR‐laden nanoporters and CD47 antibodies	Macrophages	In situ editing generates CAR‐M	GBM	[119]
VLPs	Cas9 mRNA and a Vegfa‐targeting guide RNA	Retinal pigment epithelium (RPE) cells	Knock out 44% of Vegfa in the retinal pigment epithelium and reduced the area of choroidal neovascularization by 63%	Age‐related macular degeneration (AMD)	[120]
LNPs	mRNA	HSCs	Eliminate defective sickle‐shaped blood cells	Genetic disorders	[121]

Ex vivo engineering				
Types of carriers	Nanostructures	Therapeutic compositions	Indications	Refs.
Living cells				
T cells	Immunomagnetic beads	Anti‐CD3/CD28 CAR‐T	Solid tumors	[122]
	IL‐12‐loaded human serum albumin (HSA) nanoparticles	IL‐12 CAR‐T	Tumor‐immunotherapy	[123]
Macrophages	Doxorubicin‐loaded nanoparticles	Doxorubicin	Cancer	[124]
	Magnetic nanoparticles loaded with doxorubicin and indocyanine green	Doxorubicin and indocyanine green	Solid tumors	[125]
Neutrophils	TPZ‐loaded SiO_2_ nanoparticles	TPZ, CAR‐ neutrophils	GBM	[126]
	PTX‐loaded magnetic nanogels	PTX	GBM	[127]
	Cabazitaxel /Teriparatide‐loaded PLGA nanoparticles	Cabazitaxel /Teriparatide	Prostate cancer /Osteoporosis	[128]
Yeast cells	Nano‐CaCO3	Curcumin	Gastritis	[129]
Microalgae	Antibiotic‐loaded neutrophil membrane‐coated polymeric nanoparticles	Antibiotic	Acute bacterial pneumonia	[130]
**Cell‐derived structures**				
Cytomembrane vesicles	Cell‐derived nanovesicle (CNV)	siR‐Sox2, gelonin, and paclitaxel	Cancer	[131]
	Cellular nanovesicles	IL‐15/IL‐15R, PD‐1/PD‐L1 inhibitor	Melanoma	[132]
	Cellular nanovesicles	CD40, mycophenolate mofetil	Systemic lupus erythematosus	[133]
	Fusion nanovesicles	Oxaliplatin, Platelet‐derived membrane	Triple‐negative breast cancer	[134]
EVs	Exosomes	Anti‐human CD3 and anti‐human HER2	Breast cancer	[135]
	Exosomes	Antibodies anti‐CD3 and EGFR antibodies, PD‐1, OX40L	Triple‐negative breast cancer	[136]
	Tumor EVs	Toll‐like receptor 9 agonists	Lymphoma, melanoma	[137]
OMVs	OMVs	Tumor antigens	Lung melanoma, subcutaneous colorectal cancer	[138]
	OMVs	RNA binding protein, L7Ae, and lysosomal escape protein, listeriolysin O	Melanoma, colon cancer	[139]
	OMVs	PD‐1	Cancer	[140]
**Biomimetic self‐assembly systems**				
DNA	DNA robotic nanostructure	Antigen (peptide) and toll‐like receptor (TLR) agonists	Cancer immunotherapy	[141]
	DNA origami	CRISPR/Cas9 gene editing system, DNA aptamer and influenza hemagglutinin (HA) peptide	Cancer gene therapy	[142]
	DNA origami nanocages	Cas9 RNPs	Cancer gene therapy	[143]
Protein	Protein nanocages	Epirubicin /camptothecin	Brain tumor, metastatic liver cancers, and drug‐resistant breast tumor	[144]
	Apoferritin nanocages	siRNA	Breast cancer	[145]
	Bioinspired lipoprotein system, albumin‐bound paclitaxel nanoparticles	Nitric oxide, paclitaxel	Breast cancer	[146]
Membrane‐coated NPs	T cell membrane‐encapsulated albumin nanoparticles	ORY‐1001, PD1	Breast cancer, melanoma, colon cancer	[147]
	Cell membrane‐coated BTO nanoparticles	PD‐L1 antibody, barium titanate	Melanoma	[148]
	A ceria nanoparticle‐immobilized mesenchymal stem cell nanovesicle hybrid system	Ceria, MSC	Arthritis	[149]
Nanorobots	Urease‐driven nanorobots	^131^I	Bladder cancer	[150]
	Biomimetic head/hollow tail nanorobots	DOX, AuNS	Triple‐negative breast cancer, pancreatic cancer, melanoma, and lung cancer‐derived subcutaneous tumor	[151]
Artificial cells	Artificial apoptotic cells	Itaconic acid	Acute liver failure	[152]
	Artificial erythrocytes	Superoxide dismutase, catalase, carbonic anhydrase	Scavenge harmful oxygen radicals	[153]
	Artificial M2 macrophage (AM2 M) with yolk‐shell structure	Chondroitin sulfate, M2 macrophages	Osteoarthritis	[154]
	DC membrane coated polymeric nanoparticles	Anti‐CD3ε antibody, imiquimod, PD1	Cancer immunotherapy	[155]

## 9 Nanotechnology‐Mediated in Vivo Cell Engineering

**FIGURE 3:**
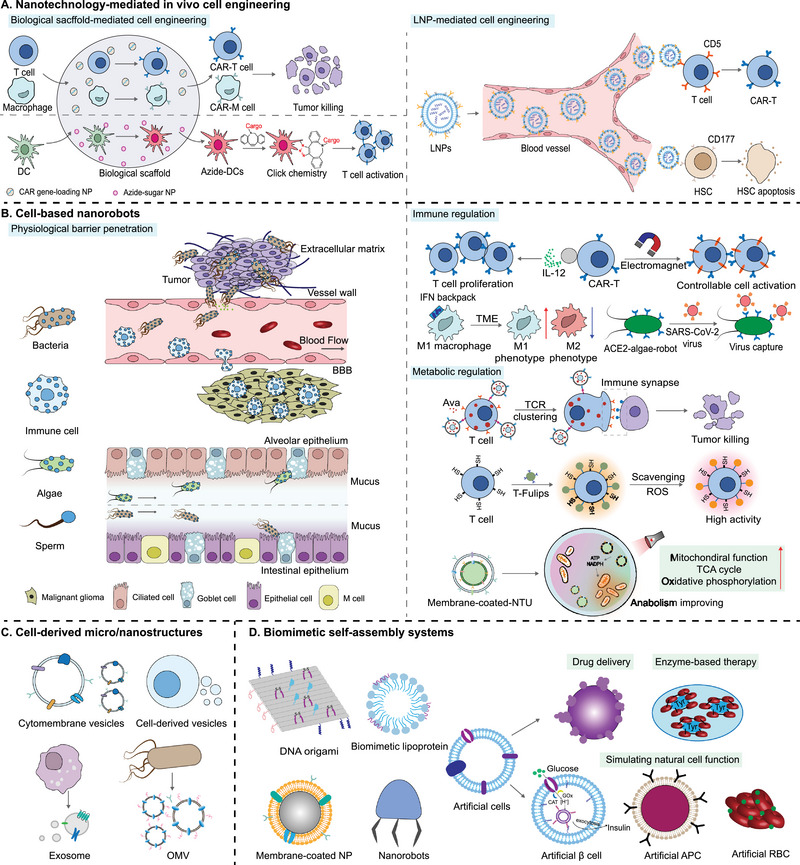
Cross‐disciplinary of synthetic biology and nanotechnology in drug delivery. (A) Nanotechnology‐mediated in vivo cell engineering. Biological scaffold (left) and lipid nanoparticles (LNPs) (right) are the most studied nanotechnology‐based in vivo cell engineering strategies. (B) Cell‐based nanorobots. Cell‐based nanorobots can navigate through intricate physiological barriers (left) and exert immune regulation and metabolic functions (right) at the site of disease. (C) Cell‐derived micro/nanostructures for drug delivery. (D) Biomimetic self‐assembly systems. DNA self‐assemblers, biomimetic lipoproteins, cell membrane‐coated nanoparticles, nanorobots and artificial cells have been studied for biomedical applications. Artificial cells, as a representation of the convergence of synthetic biology and nanotechnology, have been utilized for drug delivery, enzyme‐mediated therapy, and replacement of natural cell functions. Nanothylakoid units (NTUs); Tricarboxylic acid (TCA).

The advancement of nanotechnology provides more options for in vivo cell engineering [156] (Figure 3A). Specifically, the use of nano‐materials to construct biological scaffolds offers a stable environment for molecules and cells. It allows for the control of the activity of host immune cells by recruiting them into scaffolds. For example, nano‐porous silicon rod scaffolds filled with IL‐2 and T‐cell‐activating antibodies provide continuous stimulation of cargo T‐cells, resulting in exponential expansion of T‐cells and maintaining high activity for a prolonged period of time [117]. Similarly, DC membrane‐encapsulated scaffolds allow antigen‐specific T cell expansion, providing a scalable, modular, and customizable platform for rapid access to high‐performance T cells [157]. Incorporating nanocarriers carrying CAR genes into the Multifunctional Alginate Scaffold for T Cell Engineering and Release (MASTER) enables tight integration of the nanocarriers with the cells, expediting the reprogramming of T cells and significantly reducing the preparation time for CAR‐T cells [118]. Macrophages are the most abundant immune cells within and around glioblastoma multiforme (GBM) [158, 159, 160]. Researchers have developed an injectable hydrogel to co‐deliver macrophage‐targeting nanocarriers and CD47 antibodies into the postoperative tumor cavity of GBM, enabling in situ editing of local macrophages to generate CAR‐M capable of targeting and eliminating glioblastoma stem cells (GSCs) [119]. These nanomaterial‐based scaffolds provide a novel approach for in vivo editing of immune cells, potentially expanding the application of CAR‐based cell therapy. Furthermore, biocompatible scaffolds loading with azide‐functionalized monosaccharide (Ac_4_ManNAz) nanoparticles can effectively recruit and modulate DCs in vivo [161]. Antigen loading and co‐delivery of IL‐15 can be achieved through click chemistry to activate their antigen presentation and T cell activation capabilities. Recently, the hardness and other properties of tumors have been identified as barriers to the function of immune cells [162, 163]. Therefore, the development of scaffolds using nanomaterials to regulate tumor mechanical stress may provide a new approach for engineering cell remodeling and activity in vivo.

Compared to the complex and time‐consuming ex vivo engineering process, nanotechnology has facilitated in situ genetic engineering of host cells, overcoming problems such as moderate targeting of adoptive transferred cells. Commonly used lentiviral and adenoviral vectors have been used for in vivo T cell, B cell, and macrophage modification [164, 165, 166]. However, random integration of the viral genome greatly limits the effectiveness and safety of viral vectors. Virus‐like particles (VLPs) are nanostructures composed of viral proteins that carry molecular cargo. They may be safer than virus as they do not contain virus genetic messages. For example, researchers used VLP to deliver Cas9 mRNA and gRNA to enable in situ gene editing of ocular cells to treat viral keratitis and age‐related macular degeneration [120¸ 167]. Engineered VLPs (eVLPs), obtained by modulating glycoprotein ratios, also allow gene editing of cells in vivo [168, 169]. Coupling acid‐sensitive materials on VLPs results in significantly longer blood circulation and enhanced gene delivery efficiency to weak acidic lesions, and achieves significant therapeutic effects for cancer and rheumatoid arthritis [170]. For precise targeting of cells of interest, the use of cell membrane‐encapsulated VLPs allows selective transient delivery of genome editing mechanisms to T cells using predictable antibody‐antigen interactions [171]. To further reduce the possible side effects of VLPs, liposomes and lipid nanoparticles (LNPs) are gradually being developed for in vivo cell engineering. By regulating the surface charge of liposomes, it is possible to precisely and effectively target different organs and even cells [172, 173]. For example, intravenous injection of RNA‐lipid complex (RNA‐LPX) can precisely target DCs and macrophages in lymph nodes for efficient uptake and expression of encoded antigens [174]. Modification of targeting ligands on liposome surfaces also enables precise cell targeting and genetic engineering. For example, loading CD5 antibodies on liposomes allows the T cell targeting CAR expression that clears activated cardiac fibroblasts [175]. LNPs with CD177 antibodies recognize receptors on hematopoietic stem cells (HSCs) and increase the proportion of functional hemoglobin to 91.7%, eliminating defective sickle‐shaped blood cells [121]. This strategy of directly replacing diseased cells with modified cells expands the use of gene therapy and provides new insights into in vivo cell engineering.

## 10 Cell‐Based Nanorobots

One significant challenge in the application of nanomaterials and engineered cells within the body is overcoming biological barriers and resistance in diseased tissues, which hinders the delivery of therapeutic agents to target cells. Table 3 summarizes the current methods of drug delivery used to cross physiological barriers and their advantages and shortcomings. Cell‐based nanorobots, driven by biological components or physical fields, integrate therapeutic sources and control systems into a complete system. They are powerful biohybrid systems formed by the covalent coupling or internalization of nanomaterials with cells. These biohybrid cell‐based nanorobots show outstanding advantages in penetrating biological barriers and regulating functions with the integration of therapeutic drugs and controllable elements into a complete system (Figure 3B).

**TABLE 3:** Strategies to overcome physiological obstacles and their advantages and shortcomings.

Strategies	Examples	Advantages	Shortcomings	Refs.
Nanoparticulate systems	Liposomes	Biocompatible, modifiable, non‐immunogenic	Easily cleared in the body	[176, 177]
	Lipid nanoparticles	Easy surface modification, good loading of lipophilic drugs	High toxicity	[178, 179]
	Polymeric nanoparticles	Biodegradable, biocompatible	Increased risk of particle aggregation and toxicity	[180, 181, 182]
	Exosomes	Penetrate the BBB, low toxicity and high biocompatibility	Difficult to mass produce, only exists in the research stage	[177, 183, 184]
Cell‐based nanorobots	Cell‐based nanorobots modified with bacterial flagella	Bacterial flagella sense environmental stimuli and generate directed propulsion		[185, 186, 187]
	Sperm cell‐based robots	Capable of self‐propulsion and navigation		[188, 189]
	Doxorubicin‐loaded nanoparticles on macrophage	Traverse the vascular wall and extracellular matrix, reduce the toxicity of nanoparticles to cells		[124, 125]
	A neutrophil‐based microrobot	Cross the blood‐brain barrier through the positive chemotactic motion	Immune attack and clearance	[126, 128]
	Artificial‐enzyme‐modified bifidobacterium longum probiotics	Penetrate the intestinal mucus barrier, persistently scavenge elevated ROS		[190]

The blood flow, vascular wall, extracellular matrix, blood‐brain barrier (BBB), marrow‐blood barrier (MBB), gastrointestinal mucosa, etc. restrict the target delivery of drugs and engineered cells, limiting their therapeutic effects. Considering that cell‐based nanorobots have unique chemotactic mobility and can be remotely controlled, they can navigate therapeutic agents through physiological barriers and deliver them to the disease site as desired. This strategy of targeting disease sites with nanoparticles along with cells is also known as cell hitchhiking or cell backpack strategy. For example, bacterial flagella can sense environmental stimuli and generate directed propulsion [191]. Cell‐based nanorobots modified with bacterial flagella utilize their environmental sensing and driving capabilities to overcome blood flow obstacles [185, 186, 187]. Sperm flagella demonstrate similar abilities in combating blood flow [192]. By combining with magnetic nanomaterials, sperm cell‐based robots can actively counteract blood flow and deliver heparin and chemotherapy drugs to target tissues as needed [188, 189]. The vascular wall and extracellular matrix are the most direct barriers that limit the targeted delivery of therapeutic drugs from the systemic circulation to diseased cells. Fortunately, nanoparticle‐loaded bacteria can penetrate the vascular wall and extracellular matrix barrier through a drilling mechanism guided by chemical inducers and magnetic fields [193]. Macrophages loaded with nanodrugs can traverse the vascular wall and extracellular matrix without disrupting the blood vessels under the unique ability of leukocytes to migrate across blood vessels [124, 125]. BBB is an important barrier that protects the brain from toxins and pathogens, but it also limits the passage of drugs. Numerous studies have shown that neutrophils can traverse BBB along gradients of inflammatory factors. Therefore, neutrophils are being continually developed as drug‐delivery vehicles to target brain tumors [194, 195, 196]. Recently, researchers have developed neutrophil‐based nanorobots (neutrobots) for actively migrating to the brain and crossing BBB under the dual action of magnetic fields and chemokines [127]. Neutrobots are considered to be a promising non‐invasive brain tumor treatment technology due to the brain targeting of drugs under low‐intensity magnetic fields. To further improve its tumor‐killing ability, CAR‐neutrophils were generated to deliver Tirapazamine (TPZ)‐loaded nanoparticles, significantly inhibiting brain tumor growth [126]. Similarly, MBB limits the complete cure of orthopedic diseases. Interestingly, senescent neutrophils gained bone marrow migratory capacity [197, 198, 199]. Senescent neutrophil‐based nanorobots can deliver drugs to the bone marrow under the influence of CXCR4/CXCL12 signaling for the treatment of bone metastatic cancers and osteoporosis [128]. However, due to the difficulties of neutrophil isolation, short life span, and complex drug loading process in vitro modification, the researchers designed an in situ hitchhiking strategy to achieve drug delivery. For example, liposomes loaded with paclitaxel (PTX) target disease sites with neutrophils after injection in vivo to inhibit the recurrence of glioma in mice after surgical removal [200]. Liposomes containing anti‐Ly6G antibodies are able to target neutrophils in the bloodstream to deliver the Doxorubicin (DOX) and the non‐nucleotide stimulator of interferon genes (STING) agonist SR‐717 for post‐operative tumor treatment [201]. Other teams have reported a nano‐pathogenoid (NPN) system that can hitchhike circulating neutrophils in situ. NPN is coated with OMV secreted by bacteria, thus retaining features associated with the native bacterial pathogen and being easily recognized and internalized effectively by neutrophils. Studies have shown that after neutrophils reach the tumor site, NPN can be rapidly released under inflammatory stimulation to play an anti‐tumor role. Even more interesting, the study found that cisplatin‐loaded NPNs combined with photothermal therapy (PTT) therapy completely eradicated tumors [202]. In addition to neutrophils, nanoparticles can also be transported to disease sites by other cells. For example, researchers synthesized DOX·HCl loaded matrix metalloproteinase 2 (MMP‐2) peptide liposomes (D@MLL), which can hitchhike on circulating monocytes with the help of low‐dose radiotherapy to target intracranial GBMs sites for drug release [203]. Using the natural behavior of macrophages for apoptotic body phagocytosis and tumor homing, researchers designed an apoptotic body (AB) coated with CpG immunoadjuvant‐modified gold–silver nanorods (AuNR‐CpG/AB). Studies found that AuNR‐CpG/AB can be specifically phagocytic by monocytes and actively infiltrate into the tumor center [204]. Mucus extensively covers the surface of epithelial cells in the gastrointestinal tract and trachea, greatly limiting the bioavailability of the drug for oral or tracheal administration. Considering the wide distribution of intestinal flora, yeast armed with glucose oxidase and catalase and Bifidobacterium longum armed with artificial enzymes were designed to penetrate the intestinal mucus barrier for further delivery of therapeutics [129, 190]. In addition, algae have emerged as strong candidates for the development of nanorobots due to their cell compatibility and excellent adaptability and mobility in aquatic environments. Nanorobots based on microalgae and cyanobacteria demonstrate remarkable locomotion capabilities in pulmonary and intestinal fluids, penetrating mucus barriers to deliver drugs directly to diseased sites [130, 205]. In brief, cell‐based nanorobots provide unique penetration capabilities, opening up a fascinating path in the field of active targeting delivery.

Abnormal physiological reactions create a favorable environment for disease progression but hinder the optimal functioning of immune cells, thereby limiting disease treatment. Cell‐based nanorobots have the ability to self‐regulate in complex internal environments, eliminating the detrimental effects and maintaining cellular homeostasis. Immune magnetic bead‐modified CAR‐T (M‐CAR‐T) nanorobots, driven by magnetic acoustics, exhibit enhanced tumor infiltration and immune activation [122]. Coupling CAR‐T with microenvironment modulators enables solid tumor immunotherapy through microenvironment remodeling [123, 206, 207]. To overcome immune suppression, reprogramming tumor‐associated macrophages (TAMs) into M1 phenotype is necessary. Loading IFN‐γ backpacks onto macrophage surfaces proves to be an effective strategy [208]. Macrophage robots loaded with superparamagnetic nanoparticles (HION@Macs) effectively counteract immune suppression and generate large amounts of inflammatory cytokines, polarizing TAMs towards M1 phenotype [209]. In addition, some cell‐based nanorobots can directly identify and capture pathogens, eliminating the generation of immune suppression at its source. For example, microalgae robots effectively capture and eliminate viruses in water by chemically coupling angiotensin‐converting enzyme 2 (ACE2) receptors [210, 211]. Nanorobots based on *E. coli* actively recognize damaged cells through lectin‐mannose coupling and slowly release drugs to repair injured tissues [212].

In addition to reversing immune‐suppressive microenvironments, restoring abnormal metabolic pathways in cells is also an improvement strategy because cell metabolism is closely related to immune function. For example, the abnormal cholesterol metabolism in T cells limits their anti‐tumor activity [213, 214]. Liposomes anchored with loaded avemarib can continuously increase cholesterol levels in the T cell membrane, promoting rapid aggregation of T cell receptors (TCR) and increasing the long‐term vitality of T cells [215]. ROS scavengers displayed on the surface of T cells by targeting liposomes not only provide magnetic manipulation capabilities but also capture surface ROS on T cells, reversing T cell functional exhaustion [216]. The imbalance of cellular energy metabolism in pathological conditions is also considered the cause of disease progression [217]. However, directly providing exogenous ATP or NADPH has minimal impact on cell metabolism [218, 219]. In a creative approach, researchers transported plant thylakoids into chondrocytes and achieved photosynthesis in mammalian cells [220]. Under light exposure, the energy metabolism and amino acid metabolism of pathologically affected cells were reprogrammed, leading to restoration of cellular function and effective relief from osteoarthritis. This study constructed an independent photosynthetic system within animal cells, demonstrating the potential for cross‐species transplantation of plant‐derived systems.

## 11 Cell‐Derived Micro/Nanostructures

Cell‐derived micro/nanostructures refer to biologically functional nanomaterials obtained from bacteria or cells. These structures inherit functional characteristics of living cells while also acquiring new functionalities through synthetic biology modifications. They show better targeted towards lesions and immune regulation, making them more suitable as delivery carriers for drugs and vaccines. Here, we introduce significant advancements in cell‐derived micro/nanostructures based on synthetic biology, including cytomembrane vesicles, extracellular vesicles (EVs), and bacterial OMVs (Figure 3C).

### 11.1 Cytomembrane Vesicles

Cytomembrane vesicles are obtained from cells after hypotonic solution, mechanical membrane disruption, sucrose gradient centrifugation, and mechanical extrusion [221]. These vesicles exhibit excellent biocompatibility and stability, making them suitable for encapsulating therapeutic drugs [222]. However, challenges such as inadequate targeting ability, low intracellular delivery efficiency, and weak immune modulation capabilities have hindered their applications [223]. The flourishing field of synthetic biology provides powerful tools to address these issues. For instance, engineered cytomembrane vesicles (eFT‐CNVs) effectively bind to GPC3‐overexpressing cancer cells and undergo fusogen‐mediated membrane fusion, enabling cytoplasmic drug delivery [131]. Expressing immune checkpoint antibodies or receptors on the cell membrane, such as PD‐1 single‐chain antibody (PD‐1 scFv) and PD‐1 protein, not only enhances the targeting ability but also competitively blocks the PD‐1/PD‐L1 immune checkpoint axis, restoring immune cell function [224, 225, 226]. Expression of immunostimulants on cytomembrane vesicles can regulate immune responses. For example, cytomembrane vesicles expressing IL‐15/IL‐15Rα complex effectively promote the proliferation and activation of CD8^+^ T cells, serving as potent candidates for nano‐vaccines [132, 227]. Stable expression of CD40 protein on cytomembrane vesicles can target CD4^+^ T cells and inhibit B cell proliferation to treat systemic lupus erythematosus (SLE) through CD40/CD40L interaction blockade [133]. In addition, the fusion of different cell membranes can endow cytomembrane vesicles with dual functions of targeting and immune regulation. The fusion of cell membranes expressing TIGIT and platelet cell membranes (TPNVs) effectively targets tumor residual lesions after surgery, while blocking the CD155/TIGIT pathway, providing an important strategy for postoperative consolidation therapy for tumors [134]. The fusion of cell membranes expressing ACE2 receptor and high‐expression cytokine receptors effectively adsorbs and neutralizes SARS‐CoV‐2 virus, as well as the cytokine storm caused by SARS‐CoV‐2 infection [228]. In short, modifying cells with synthetic biology and then nanosizing their cytomembranes by nanotechnology enables the construction of cytomembrane vesicles with special functions, greatly advancing the development of the field.

### 11.2 EVs

EVs, including apoptotic bodies, microvesicles, and exosomes, are composed of one or more lipid membrane‐enclosed small compartments carrying a large number of biomolecules from parent cells [229, 230]. When EVs bind to recipient cells, they deliver “cargo” into the recipient cells, thereby mediating intercellular signal communication and substance exchange [231, 232]. Engineered EVs through synthetic biology can serve as superior targeting delivery systems for precision therapy. For example, synthetic multivalent antibody retargeted exosomes (SMART‐Exos) were designed to target T cell CD3 receptors and breast cancer‐associated HER2 receptors [135]. To further improve the immunomodulatory ability, the team developed multifunctional immune‐modulating exosomes (GEMINI‐Exos) [136]. GEMINI‐Exos carried specific monoclonal antibodies against CD3 and epidermal growth factor receptor (EGFR) as well as PD‐1 and OX40L. The resulting αCD3‐αEGFR‐PD‐1‐OX40L GEMINI‐Exos induced and maintained strong anti‐tumor immunity and produced highly effective inhibition of tumors. In addition, engineered EVs are also an ideal carrier for tumor vaccines, as they can directly target DCs and activate anti‐tumor immunity [137, 233, 234]. For autoimmune diseases, engineered EVs also act as scavengers to clear over‐activated immune cells and maintain immune homeostasis [235]. Briefly, EVs engineered by synthetic biology have strong operability and practicality and can meet the therapeutic needs of different diseases.

### 11.3 OMVs

OMVs are natural vesicles produced by Gram‐negative bacteria containing pathogen‐associated molecular patterns (PAMPs). They are often used as vaccines for pathogenic microorganisms because of their superior immune activation ability [236]. Through synthetic biology modification, antigens from various viruses can be displayed on OMVs of *E. coli*., thereby preventing viral infections [237, 238, 239]. In the field of cancer immunotherapy, engineered OMVs also show promising potential for translation [240]. Using synthetic biology technology, tumor antigen peptides, RNA‐binding proteins, and PD‐1 proteins can be displayed on the surface of OMVs for the development of personalized cancer vaccines and the delivery of mRNA vaccines [138, 139, 140]. Recent research has shown that OMVs can directly train innate immune cells, enhancing their sensitivity to tumor vaccines [241]. This not only deepens our understanding of the systemic immune effects of OMVs but also advances the clinical application of engineered OMV tumor vaccines.

## 12 Biomimetic Self‐Assembly Systems

Through clever design, biomolecules and nanomaterials can self‐assemble into integrated systems that mimic certain life activities in vivo, such as DNA self‐assemblers, protein nanocages and biomimetic lipoproteins, cell membrane‐coated nanoparticles, nanorobots, and artificial cells. These biomimetic self‐assembling systems greatly enrich our understanding of the synthesis of artificial life forms and hold great significance in future research of synthetic biology and nanotechnology (Figure 3D).

### 12.1 DNA Self‐Assemblers

Nucleic acids are carriers of genetic information. Their self‐assembled structures can also be applied in the field of drug delivery [34, 35]. DNA is a natural building block with the potential for nanofabrication through highly rigorous base pairing [242, 243, 244]. DNA self‐assembly technologies mainly include DNA tile, origami, and brick assembly [245]. DNA origami allows the construction of highly complex nano‐patterns or structures by complementing a long single strand of DNA with a series of designed short DNA fragments [246]. In contrast, DNA brick does not require any long single strands, so it has modular structures, and each brick can be added or removed individually [247, 248]. Both of them are important milestones in the field of DNA self‐assembly for drug and vaccine delivery. For example, DNA origami resembling those of the human immunodeficiency virus, with surfaces covered in HIV proteins or antigens, can trigger robust immune responses [36]. Tubular DNA nanodevice vaccines effectively deliver antigens and adjuvants to the tumor sites to trigger a strong immune response [249]. Furthermore, DNA nanorobots can accurately deliver thrombin to tumors [141]. By recognizing the nucleus of endothelial cells, DNA origami undergoes structural changes and releases thrombin, thereby inhibiting tumor blood supply. DNA self‐assemblers have a large surface area, providing numerous binding sites for the Cas9 protein, making it a potential delivery vehicle for in vivo gene editing. For example, assembly guided by protospacer‐adjacent motif (PAM) can recruit and load sgRNA/Cas9 onto DNA origami, significantly downregulating the expression of tumor‐related genes [142]. Using DNA nanocages also allows for fine‐tuning the stability of encapsulated Cas9 protein, achieving the goal of gene editing [143].

### 12.2 Protein Nanocages and Biomimetic Lipoproteins

The self‐assembled structure of proteins refers to the supramolecular structure with a specific shape and function formed by the non‐covalent interaction of protein molecules in an ordered manner [250]. Protein nanocages are highly symmetrical core‐shell structures formed by the self‐assembly of subunits, which can be used as drug delivery carriers. Ferritin nanocages (FNs) have been widely used in tumor drug delivery due to their ability to target transferrin receptor 1 (TfR1) [144, 251]. In addition, the construction of FNs with a positive inner cavity (HFN+) can effectively bind nucleic acids through electrostatic adsorption, delivering nucleic acid drugs including Toll‐like receptor (TLR) nucleic acid ligands and siRNA [145, 252, 253]. Lipoproteins have been considered a promising therapeutic strategy due to their inherent blood‐brain barrier permeability and homing ability to injured tissues [254]. However, their widespread application has been hindered by complex purification, sensitivity to oxygen, and uncontrollable quality standards [255]. The use of biomimetic lipoproteins formed by self‐assembly of phospholipids and lipoprotein peptides effectively circumvents these drawbacks, making it a novel drug delivery carrier. For example, biomimetic lipoproteins loaded with photosensitizers or NO donors can remodel the tumor stroma barrier under light or NO exposure, enhancing the intratumoral infiltration of cytotoxic T lymphocytes (CTL) [146, 256, 257].

### 12.3 Cell‐Membrane Coated Nanoparticles

Due to the excellent biocompatibility of cell membranes, biomimetic nanomedicines made with cell membrane‐encapsulated drug‐carrying nanoparticles can effectively avoid clearance by the reticuloendothelial system, thereby extending their circulation time in the bloodstream [258]. Compared to cytomembrane vesicles, biomimetic nanoparticles have a core that can stably carry drugs. The core's environmental responsiveness gives the biomimetic nanoparticles controllable drug‐release properties. By combining cell membrane outer coating with synthetic biology modification, the goal of precise delivery and intelligent drug release is achieved. For example, PD‐1 overexpressing T cell membrane‐encapsulated albumin nanoparticles (OPEN) target PDL1‐expressing tumors, followed by triggering internalization of OPEN and immune checkpoint proteins [147]. IFN inducers are released from OPEN in the presence of intracellular GSH, stimulating tumor secretion of IFN. PD‐L1 antibody‐expressing biomimetic nanoparticles release antibodies in response to metal‐matrix protease 2 (MMP2) in the TME and induce tumor cell immunogenic death (ICD) [148]. Biomimetic nanoparticles (Ce‐MSCNV) composed of cerium dioxide nanoparticles and mesenchymal stem cell (MSC) membranes not only scavenge excess ROS but also promote chondrocyte repair, emphasizing the importance of synergistic action of anti‐inflammatory catalysts and immunomodulators in the treatment of inflammation [149]. Recently, researchers have developed a modularly modifiable platform of cell membrane‐coated nanoparticles based on synthetic biology [259]. They introduced new functionalities using SpyCatcher‐SpyTag binding pairs to attach multiple components to the nanoparticle surface. This modular functionalization approach simplifies the design process. In the future, a vast library of modular components can be developed, and nanoparticles encapsulated in cell membranes from various sources can be easily modified to meet specific needs.

### 12.4 Nanorobots

Unlike cell‐based nanorobots, nanorobots are microscopic machines built using nanomaterials that mimic the way cells move. They are also capable of directional propulsion in response to physical fields, chemical energy, and enzyme catalysis and have the potential for in vivo tracing and drug delivery [260, 261]. Overcoming high‐speed blood flow and complex matrix barriers is a major challenge for nanorobots to penetrate deep into diseased tissue. Researchers have developed a tardigrade‐like medical nanorobot that mimics the way tardigrades use their claws to move in dynamic environments [262]. Such nanorobots can move at high speeds in blood flow and reside on the surface of biological tissues and drug release under the influence of magnetic fields. Urease‐driven nanorobots can break down the extracellular matrix of tumors by raising the local pH through spontaneous chemical reactions [150]. This phenomenon facilitates penetration into bladder tumor tissue and favors preferential accumulation in tumor tissue. In addition, biomimetic head/hollow tail nanorobots loaded with photosensitizers can penetrate and remodel the tumor stromal barrier in the presence of a needle‐like shell and near‐infrared light [151]. Although these nanorobots have demonstrated promising initial results in drug delivery, further evaluation is needed to assess their disease‐specific efficacy and in vivo biosafety in more comprehensive studies.

### 12.5 Artificial Cells

Artificial cells are structures designed and prepared to resemble biological cells using natural or synthetic materials. Through rational design and assembly, artificial cells have made significant contributions to the treatment of disease. The ethical requirements for artificial cell experiments are simplified compared to natural cell experiments due to the large volume and wide range of materials required for artificial cell construction, which highlights the great significance of artificial cell research. Firstly, artificial cells can be used for drug delivery. Such as the design and synthesis of itaconic acid‐loaded artificial apoptotic cells (AI‐Cells) [152]. The cells can be localized in the liver and further transported to liver macrophages for precise delivery of itaconic acid. Other researchers have reprogrammed DNA to build synthetic blood cells [263]. These synthetic blood cells can be used to precisely target disease sites without affecting normal tissues by adding magnetic particles to external magnets. Secondly, artificial cells can replace the functions of natural cells. Artificial erythrocytes (PolyHb‐SOD‐CAT‐CA) enable gas exchange between the organism and the outside world while scavenging harmful oxygen radicals [153]. These artificial cells have been shown in animal experiments to be significantly superior to whole blood in a number of metrics, such as restoration of the ST segment of the electrocardiogram and reduction of lactate levels. Artificial β cells sense high glucose in the environment, initiate programmable insulin gene transcription and protein translation and secrete insulin [45]. Metal‐organic framework (MOF)‐based artificial neutrophils target inflammation and produce hypochlorous acid, showing great potential in tumor therapy [264]. Artificial M2 macrophages loaded with gelatin and chondroitin sulphate (ChS) target areas of inflammation and achieve burst release of ChS during acute episodes of inflammation, alleviating inflammatory progression [154]. Artificial antigen presenting cells (aAPCs) constructed using engineered dendritic cell membranes or tumor cell membranes directly target and stimulate T cells, omitting the need for APCs to process presenting antigens, and exhibit superior T cell activation efficiency [155, 265, 266]. Thirdly, artificial cells can be used for enzyme‐based therapy. For example, artificial cells containing tyrosinase can enter melanoma cells and thus inhibit the growth of skin cancer [267]. Artificial erythrocytes encapsulating L‐methionine lyase are cytotoxic to many human cancer cell lines while remaining non‐toxic to normal cells [268]. In conclusion, the application of artificial cells in the field of biomedicine has shown significant value, providing great imagination for the future medical model of mankind.

## 13 Ai for Synthetic Biology

**FIGURE 4:**
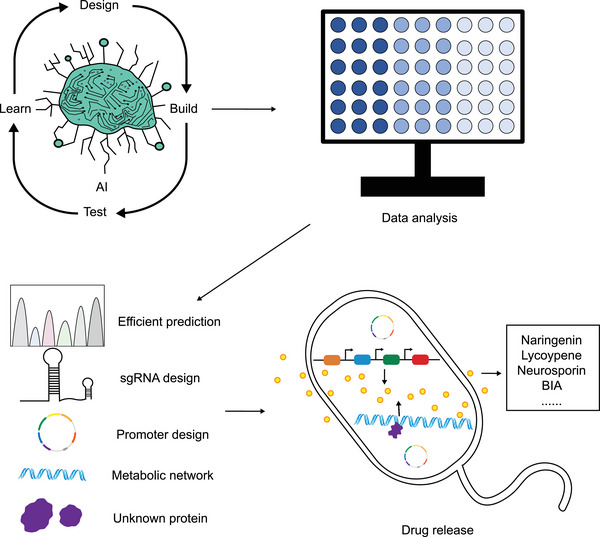
AI for synthetic biology. AI can improve synthetic biological tools based on the ability of continuous learning and the intelligent detection ability of unknown space to enhance the delivery and release capacity of engineered cells.

Artificial Intelligence (AI) technology provides powerful analytical tools and simulation methods for various fields based on the continuous learning ability of massive data and intelligent exploration ability in unknown space [269]. Applying AI to synthetic biology will help predict and optimize cell behavior and delivery mechanisms. AI can optimize synthetic biology tools to engineer cells more accurately (Figure 4). Gene editing technology is the main tool to achieve engineered cells, and base editing systems can achieve precise editing with single nucleotide accuracy. However, deaminase, the core component of the base editing system, comes from a single family, resulting in a few types of base editors, which makes it difficult to meet the needs of diversified editing [270, 271]. Therefore, it is particularly important to use AI to help discover new deaminase to develop a new base editor. AlphaFold2 is a protein structure prediction system developed by the Deepmind team [272]. The researchers identified 45 single‐stranded cytosine deaminases (Sdd) and 13 double‐stranded cytosine deaminases (Ddd) using AlphaFold2, and further developed a novel Sdd6‐cytosine base editor (CBE), with an editing efficiency of up to 43.1% in mouse cell lines [273]. Other researchers have used AI techniques to predict the editing effects of base editors. For instance, the researchers built a deep learning model algorithm, CGBE‐SMART, which can accurately predict the single‐base editing efficiency and editing effect of the novel optimized C‐to‐G base editor (OPTI‐CGBEs) [274]. The researchers also performed extensive analysis of adenine and cytosine base editors on libraries containing 28,294 lentiviral integrated gene sequences, and built BE‐DICT, an attention‐based deep learning algorithm to predict base editing outcomes with high accuracy [275]. At the same time, AI can guide the optimization of RNA (gRNA) design in CRISPR‐Cas9 technology [276]. Applying molecular dynamics (MD) simulations to computational analysis of CRISPR systems, the researchers proposed the first CRISPR targeting effect prediction and sgRNA optimization model CRISOT based on molecular interaction fingerprints. The results showed that CRISOT successfully optimized sgRNA targeting PCSK9, a key gene for primary hypercholesterolemia, and BCL11A, a key gene for sickle cell anemia, and improved its targeting specificity, indicating that CRISOT is an effective and scalable system for genome‐wide CRISPR off‐target prediction and sgRNA optimization [277].

AI also has a wide range of applications in cellular metabolic engineering. While existing metabolic engineering trial‐and‐error methods are time‐consuming and laborious, machine learning (ML) can analyze large amounts of biological data to provide accurate predictions in genetic circuit design, pathway design and optimization, and screening for high‐yielding cells [278]. As a key element in regulating gene expression at the transcriptional level, promoters are an important consideration in the design of synthetic circuits and metabolic engineering [279, 280, 281]. ML can be used to design and optimize promoter sequences to regulate gene expression. In one study, the team developed the ProEnsemble machine learning framework to optimize evolutionary pathway promoter combinations to significantly enhance pathway flux. Through this strategy, naringenin pathway enzymes evolved in parallel along a predictable trajectory in 6 weeks, and the final naringenin titer reached 3.65 g·L^−1^ in batch fermentation, which exceeded the value reported in the previous literature [280]. Researchers also reported an integrated robotic platform based on machine learning algorithms, BioAutoMata, to fully automate the design, build, test, and learn (DBTL) process for biosystems design and demonstrated it in optimizing the lycopene biosynthesis pathway. Experiments showed that by fine‐tuning the expression of genes involved in its biosynthesis, BioAutomata could screen out mutant *E. coli* strains with a lycopene titer 1.77 times higher [282]. In another application, the researchers developed an active and machine learning method (ActiveOpt) that can identify ribosome binding sites (RBSs) and gene combinations with higher valine and neurosporin yields in fewer experiments, resulting in high‐yield strains for more efficient drug delivery [283]. In addition, AI can also discover unknown enzymes in metabolic engineering to achieve the mass production of certain chemicals in living organisms. For example, by building a machine learning prediction model, the researchers successfully discovered the enzyme that produces the precursor chemical benzylisoquinoline alkaloid (BIA) for opioid analgesics to achieve the production of BIA using *E. coli* [284].

In general, AI has a great role in promoting gene editing efficiency and metabolic pathway optimization in synthetic biology. Accordingly, engineered cells based on synthetic biology strategies will also be improved, allowing cells to express higher levels of drugs to improve their drug delivery efficiency.

## 14 Conclusions and Perspectives

**FIGURE 5:**
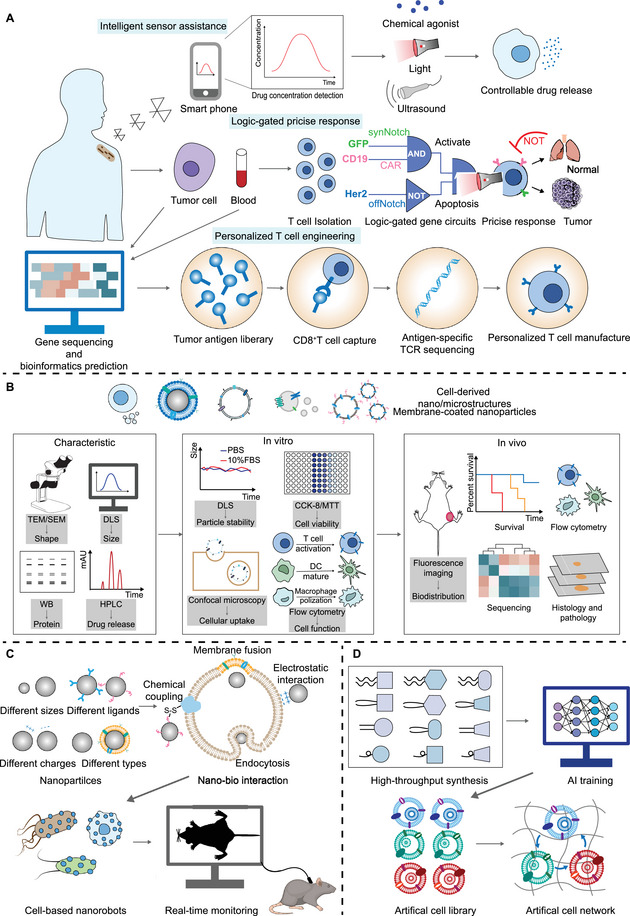
Potential strategies to tackle limitations of synthetic biology‐inspired cells and promote the cross‐application with nanotechnology. (A) Assisted by intelligent sensors, logic gate‐controlled precise responses, and personalized cellular engineering are three potential approaches to address the limitations of synthetic biology‐inspired therapeutic cells. (B) Overview of the common characterization methods of cell‐derived micro/nanostructures and membrane‐coated nanoparticles. The physical and chemical properties of nanostructures are first characterized. The in vitro biological effects of nanostructures correspond to their physicochemical properties. Subsequently, the in vivo distribution, efficacy and toxicity of nanostructures are analyzed using various techniques. (C) Analyze the nano‐bio interactions of nanoparticles with different properties and construct cell‐based nanorobots with optimal and stable performance based on nanoparticle and cellular properties. Monitor the movement of cell‐based nanorobots in real time using in vivo imaging equipment. (D) Biological component libraries are constructed using high‐throughput synthesis strategies. Deep computation using AI to analyze the optimal combination patterns between different biological components. Then, different artificial cells are obtained based on the results of the AI calculations. Finally, the artificial cell network is constructed and the artificial cells with different functions are connected to form a complete functional circuit. Transmission electron microscope (TEM); Scanning electron microscope (SEM); Dynamic light scattering (DLS); Western blot (WB); High performance liquid chromatography (HPLC); Cell counting kit‐8 (CCK‐8); 3‐(4,5‐dimethyl‐2‐thiazolyl)‐2,5‐diphenyl‐2‐H‐tetrazolium bromide (MTT); Phosphate buffered saline (PBS); Fetal bovine serum (FBS).

Synthetic biology‐based engineered cells have the potential to actively target disease sites and respond to disease signals, highlighting their potential in drug delivery. Despite showing great promise in changing the treatment paradigm, these cells still face many challenges to their clinical approval (Figure 5A). One key limitation is the lack of sensors that can accurately respond to disease characteristics. Most engineered cells rely on existing proteins that recognize molecules of interest. While arming these proteins with cells gives them specific targeting capabilities, they are not universal. For example, CAR‐T therapy achieves killing of blood tumors by CARs targeting CD19. However, some CD19‐negative patients still do not benefit from CAR‐T therapy. One possible solution is to design personalized antibody fragments to arm cells based on patient genetic information, allowing cells to specifically bind to the patient's personalized target molecules and greatly expand the applicability of engineered cells [285]. In addition, using the synNotch receptor to create a diverse library of multi‐receptor cell–cell recognition circuits, and then employing logic gates to control cell recognition and response to multiple antigens, also offers a powerful toolkit for precise response to disease [286]. Another challenge is the controllable output of cells. While engineered cells can produce therapeutics under specific stimuli, their secretion is unmanageable. Especially for highly toxic drugs, a strict control of drug secretion is necessary. The combination of intelligent biological sensors based on synthetic biology and wearable electronic devices is expected to detect the concentration of drugs in the body in real time and provide accurate treatment plans for patients through modular biological computing elements, ensuring that patients can adjust the level of therapeutic cell drug release through external intervention (such as light exposure [287]).

In addition, the potentially fatal side effects of engineering cells are also great obstacles to limiting their clinical applications. For example, CAR‐T cell therapy may cause cytokine release storms (CRS), which can trigger systemic inflammation, organ failure, and even death [288]. The use of IL‐6 adsorbable hydrogels or polyethylene glycolisation modified CAR‐T offers great promise for safer CAR‐T cell therapy [289, 290]. The need for personalized therapies and the cost of manufacturing engineered cells also limit their mass production [291]. The main steps in the current production of CAR‐T cell therapies include isolation and enrichment of T cells, activation of T cells, CAR gene transfer using viral or non‐viral vector systems, cell expansion in vitro, and cryopreservation of CAR‐T cells. The entire manufacturing cycle is 2–4 weeks, which greatly increases the cost problem in the manufacturing process [292]. In order to shorten the CAR‐T generation time and reduce the experimental cost, there are three main solutions. One is the rapid generation of CAR‐T in vivo using biomaterials mentioned above, such as Drydux [293], a new macroporous biomaterial scaffold constructed, and an implantable MASTER [118], which reduces the manufacturing and processing time of CAR‐T to 1–3 days. The second approach is to use allogeneic cells from healthy donors, reducing the number of steps and time required to manufacture engineered cells. But at the same time, allogeneic cells also face a new safety challenge—immune rejection, which not only makes them unable to play a therapeutic role, but may cause serious complications and even endanger the life of patients. The application of universal cells has greatly improved the immune rejection, such as through TALEN‐mediated gene editing and adeno‐associated virus (AAV)‐dependent gene insertion, the researchers have developed a novel immune escape universal CAR‐T cell scaffold that enables CAR‐T to overcome the attack of isoreactive NK cells and T cells [294]. And researchers reported an allogeneic CAR natural killer T cell (AlloCAR‐NKT) differentiated from human hematopoietic stem cells and progenitor cells. AlloCAR‐NKT cells exhibit characteristics of low graft‐versus‐host disease (GvHD) and CRS risk, with a stable “low immunogenicity” phenotype [295]. In addition, universal CAR‐T cell therapy has been used in phase I clinical trials [296]. The researchers obtained CD7/CD3/HLA‐II negative CAR T cells by gene editing technology, and found that such universal CAR T cells could resist GvHD and allogeneic rejection in vitro and in animal models. In a subsequent phase I clinical trial (NCT04538599) in 12 patients, 81.8% experienced an objective response and the complete response rate was 63.6% of the 11 evaluable patients. At the same time, the study showed that CRS was mild, and neither GvHD nor immune effector cell associated neurotoxic syndrome (ICANS) occurred. In addition to general‐purpose CAR T cells, general‐purpose tumor vaccines have been developed and demonstrated efficacy and safety in mice and rhesus monkeys [297]. The final strategy is to improve manufacturing processes to reduce the preparation time of engineered cells, such as Novartis’ T‐Charge [298] and CliniMACS Prodigy [299]. As an automated cell processing system, CliniMACS Prodigy can produce engineered cells according to uniform standards to ensure their quality and stability. Finally, ethical concerns about engineered cells are also a challenge preventing their widespread use. Due to violations of scientific ethics during the use of gene therapy in the past, such as the gene‐edited babies incident, there has been a lot of negative public opinion about cell therapy [300, 301]. Therefore, while emphasizing the superior therapeutic effects of cell therapy, ethical review of engineered cells should be strengthened to ensure that cell therapy research complies with ethical norms and seeks to minimize risks to subjects and maximize benefits.

The advancement of nanotechnology has expanded the range of synthetic biology‐based cell therapy. Nanocarriers, with their easy customization and targeting properties, significantly improve the effectiveness of in vivo cell engineering. Cell‐based nanorobots, inspired by nanotechnology and synthetic biology, along with cell‐derived micro/nanostructures and artificial self‐assembly systems, demonstrate substantial clinical potential. However, these technologies are still in the early stages, especially in terms of establishing standardized collection methods. Biomimetic drug delivery systems must undergo a series of physicochemical property characterizations and in vitro and in vivo functional assessments [115] (Figure 5B). At the cellular level, it is crucial to thoroughly investigate the safety, internalization pathways, and the impact on inflammatory pathways, programmed cell death, and functional regulation pathways of biomimetic systems. When working with animal models, it is essential to use various methods to label and detect the distribution of biomimetic systems and to comprehensively evaluate their biological functions using immunology, multi‐omics, and histopathological methods. For cell‐based nanorobots, it is important to fully investigate the interaction between nanomaterials and cells and to select the most suitable nanocomponents for multifunctional cell modification (Figure 5C). Additionally, real‐time monitoring of cell nanorobots will help to precisely control their targeting and regulatory capabilities. In the case of artificial cells, the high‐throughput synthesis of stimulus‐responsive biomaterials, the fabrication of complex‐structured artificial cells or artificial cell networks, and the use of artificial intelligence (AI) to optimize the design of artificial cells will further advance the development of artificial cells (Figure 5D).

In summary, synthetic biology‐based engineered cells are emerging as an alternative to current small molecule drugs and protein therapies, offering a versatile and controllable platform for drug delivery. In the future, various databases will continue to drive the clinical translation of engineered cells. An extensive repository of clinical samples provides a wealth of interesting targets, while a large nanoparticle information database consolidates diverse nano‐bio interactions. Furthermore, a comprehensive database of preclinical animal studies fully demonstrates therapeutic effects. Additionally, AI may have the ability to predict the optimal cell engineering strategies for a specific disease after learning from large databases. These predictions may offer insights into the rational design of engineered cells in drug delivery. The combination of advancing technologies is continually enriching the synthetic biology‐based cell toolbox, leading to more effective treatments for serious diseases.

## Author Contributions

Wenzhe Yi and Shuangshuang Hu wrote the manuscript and created the figures and tables. Wenzhe Yi, Shuangshuang Hu, Xindi Qian and Wenlu Yan revised the initial manuscript and figures. Wenzhe Yi and Yaping Li provided the conceptual idea and revised the manuscript. All authors have read and approved the final manuscript.

## Conflict of Interest Statement

The authors declare no conflicts of interest. Yaping Li is a member of the *Exploration* editorial board, and he was not involved in the handling or peer review process of this manuscript.

## References

[1] R. Langer , “Drug Delivery and Targeting,” Nature 392 (1998): 5–10. 9579855

[2] T. M. Allen and P. R. Cullis , “Drug Delivery Systems: Entering the Mainstream,” Science 303 (2004): 1818–1822. 15031496 10.1126/science.1095833

[3] M. J. Mitchell , M. M. Billingsley , R. M. Haley , M. E. Wechsler , N. A. Peppas , and R. Langer , “Engineering Precision Nanoparticles for Drug Delivery,” Nature Reviews Drug Discovery 20 (2021): 101–124. 33277608 10.1038/s41573-020-0090-8 PMC7717100

[4] J. D. Martin , H. Cabral , T. Stylianopoulos , and R. K. Jain , “Improving Cancer Immunotherapy Using Nanomedicines: Progress, Opportunities and Challenges,” Nature Reviews Clinical Oncology 17 (2020): 251–266. 10.1038/s41571-019-0308-z PMC8272676 32034288

[5] F. Farjadian , A. Ghasemi , O. Gohari , A. Roointan , M. Karimi , and M. R. Hamblin , “Nanopharmaceuticals and Nanomedicines Currently on the Market: Challenges and Opportunities,” Nanomedicine 14 (2019): 93–126. 30451076 10.2217/nnm-2018-0120 PMC6391637

[6] D. E. Cameron , C. J. Bashor , and J. J. Collins , “A Brief History of Synthetic Biology,” Nature Reviews Microbiology 12 (2014): 381–390. 24686414 10.1038/nrmicro3239

[7] F. Meng and T. Ellis , “The Second Decade of Synthetic Biology: 2010–2020,” Nature Communications 11 (2020): 5174. 10.1038/s41467-020-19092-2 PMC7560693 33057059

[8] M. P. McNerney , K. E. Doiron , T. L. Ng , T. Z. Chang , and P. A. Silver , “Theranostic Cells: Emerging Clinical Applications of Synthetic Biology,” Nature Reviews Genetics 22 (2021): 730–746. 10.1038/s41576-021-00383-3 PMC8261392 34234299

[9] A. Cubillos‐Ruiz , T. Guo , A. Sokolovska , et al., “Engineering Living Therapeutics With Synthetic Biology,” Nature Reviews Drug Discovery 20 (2021): 941–960. 34616030 10.1038/s41573-021-00285-3

[10] N. Zhao , Y. Song , X. Xie , et al., “Synthetic Biology‐Inspired Cell Engineering in Diagnosis, Treatment and Drug Development,” Signal Transduction and Targeted Therapy 8 (2023): 112. 36906608 10.1038/s41392-023-01375-x PMC10007681

[11] C. J. Bashor , I. B. Hilton , H. Bandukwala , D. M. Smith , and O. Veiseh , “Engineering the Next Generation of Cell‐Based Therapeutics,” Nature Reviews Drug Discovery 21 (2022): 655–675. 35637318 10.1038/s41573-022-00476-6 PMC9149674

[12] P. Braendstrup , B. L. Levine , and M. Ruella , “The Long Road to the First FDA‐Approved Gene Therapy: Chimeric Antigen Receptor T Cells Targeting CD19,” Cytotherapy 22 (2020): 57–69. 32014447 10.1016/j.jcyt.2019.12.004 PMC7036015

[13] W. Li , Z. Su , M. Hao , C. Ju , and C. Zhang , “Cytopharmaceuticals: An Emerging Paradigm for Drug Delivery,” Journal of Controlled Release 328 (2020): 313–324. 32889055 10.1016/j.jconrel.2020.08.063

[14] S. Ausländer , D. Ausländer , and M. Fussenegger , “Synthetic Biology—The Synthesis of Biology,” Angewandte Chemie International Edition 56 (2017): 6396–6419. 27943572 10.1002/anie.201609229

[15] S. Hirschi , T. R. Ward , W. P. Meier , D. J. Müller , and D. Fotiadis , “Synthetic Biology: Bottom‐Up Assembly of Molecular Systems,” Chemical Reviews 122 (2022): 16294–16328. 36179355 10.1021/acs.chemrev.2c00339

[16] S. Rémy , L. Tesson , S. Ménoret , C. Usal , A. M. Scharenberg , and I. Anegon , “Zinc‐Finger Nucleases: A Powerful Tool for Genetic Engineering of Animals,” Transgenic Research 19 (2010): 363–371. 19821047 10.1007/s11248-009-9323-7

[17] J. K. Joung and J. D. Sander , “TALENs: A Widely Applicable Technology for Targeted Genome Editing,” Nature Reviews Molecular Cell Biology 14 (2013): 49–55. 23169466 10.1038/nrm3486 PMC3547402

[18] R. C. Sterner and R. M. Sterner , “CAR‐T Cell Therapy: Current Limitations and Potential Strategies,” Blood Cancer Journal 11 (2021): 69. 33824268 10.1038/s41408-021-00459-7 PMC8024391

[19] F. Sinclair , A. A. Begum , C. C. Dai , I. Toth , and P. M. Moyle , “Recent Advances in the Delivery and Applications of Nonviral CRISPR/Cas9 Gene Editing,” Drug Delivery and Translational Research 13 (2023): 1500–1519. 36988873 10.1007/s13346-023-01320-z PMC10052255

[20] A. Pickar‐Oliver and C. A. Gersbach , “The Next Generation of CRISPR–Cas Technologies and Applications,” Nature Reviews Molecular Cell Biology 20 (2019): 490–507. 31147612 10.1038/s41580-019-0131-5 PMC7079207

[21] A. Katti , B. J. Diaz , C. M. Caragine , N. E. Sanjana , and L. E. Dow , “CRISPR in Cancer Biology and Therapy,” Nature Reviews Cancer 22 (2022): 259–279. 35194172 10.1038/s41568-022-00441-w

[22] Y. R. Choi , K. H. Collins , L. E. Springer , et al., “A Genome‐Engineered Bioartificial Implant for Autoregulated Anticytokine Drug Delivery,” Science Advances 7 (2021): eabj1414. 34516920 10.1126/sciadv.abj1414 PMC8442875

[23] Y. Lu , J. Xue , T. Deng , et al., “Safety and Feasibility of CRISPR‐Edited T Cells in Patients with Refractory Non‐Small‐Cell Lung Cancer,” Nature Medicine 26 (2020): 732–740. 10.1038/s41591-020-0840-5 32341578

[24] M. J. Volk , V. G. Tran , S. I. Tan , et al., “Metabolic Engineering: Methodologies and Applications,” Chemical Reviews 123 (2023): 5521–5570. 36584306 10.1021/acs.chemrev.2c00403

[25] R. Geiger , J. C. Rieckmann , T. Wolf , et al., “L‐Arginine Modulates T Cell Metabolism and Enhances Survival and Anti‐Tumor Activity,” Cell 167 (2016): 829–842. 27745970 10.1016/j.cell.2016.09.031 PMC5075284

[26] V. Bronte and P. Zanovello , “Regulation of Immune Responses by L‐Arginine Metabolism,” Nature Reviews Immunology 5 (2005): 641–654. 10.1038/nri1668 16056256

[27] X. He , H. Lin , L. Yuan , and B. Li , “Combination Therapy with L‐Arginine and α‐PD‐L1 Antibody Boosts Immune Response against Osteosarcoma in Immunocompetent Mice,” Cancer Biology & Therapy 18 (2017): 94–100. 28045576 10.1080/15384047.2016.1276136 PMC5362985

[28] F. P. Canale , C. Basso , G. Antonini , et al., “Metabolic Modulation of Tumours With Engineered Bacteria for Immunotherapy,” Nature 598 (2021): 662–666. 34616044 10.1038/s41586-021-04003-2

[29] S. Jiang , R. Wang , D. Wang , et al., “Metabolic Reprogramming and Biosensor‐Assisted Mutagenesis Screening for High‐Level Production of L‐Arginine in Escherichia Coli,” Metabolic Engineering 76 (2023): 146–157. 36758663 10.1016/j.ymben.2023.02.003

[30] N. Knödlseder , M. J. Fábrega , J. Santos‐Moreno , et al., “Delivery of a Sebum Modulator by an Engineered Skin Microbe in Mice,” Nature Biotechnology 42: 1661–1666. 10.1038/s41587-023-02072-4 38195987

[31] Y. H. Chan and S. G. Boxer , “Model Membrane Systems and Their Applications,” Current Opinion in Chemical Biology 11 (2007): 581–587. 17976391 10.1016/j.cbpa.2007.09.020 PMC2196400

[32] N. J. Gaut and K. P. Adamala , “Reconstituting Natural Cell Elements in Synthetic Cells,” Advanced Biology 5 (2021): e2000188. 33729692 10.1002/adbi.202000188

[33] Q. Wang , Z. Hu , Z. Li , T. Liu , and G. Bian , “Exploring the Application and Prospects of Synthetic Biology in Engineered Living Materials,” Advanced Materials: e2305828. 10.1002/adma.202305828 37677048

[34] A. V. Pinheiro , D. Han , W. M. Shih , and H. Yan , “Challenges and Opportunities for Structural DNA Nanotechnology,” Nature Nanotechnology 6 (2011): 763–772. 10.1038/nnano.2011.187 PMC3334823 22056726

[35] S. Dey , C. Fan , K. V. Gothelf , et al., “DNA Origami,” Nature Reviews Methods Primers 1 (2021): 13.

[36] R. Veneziano , T. J. Moyer , M. B. Stone , et al., “Role of Nanoscale Antigen Organization on B‐cell Activation Probed Using DNA Origami,” Nature Nanotechnology 15 (2020): 716–723. 10.1038/s41565-020-0719-0 PMC7415668 32601450

[37] X. Wu , C. Yang , H. Wang , et al., “Genetically Encoded DNA Origami for Gene Therapy in Vivo,” Journal of the American Chemical Society 145 (2023): 9343–9353. 37070733 10.1021/jacs.3c02756

[38] C. Gu , T. Zhang , C. Lv , Y. Liu , Y. Wang , and G. Zhao , “His‐Mediated Reversible Self‐Assembly of Ferritin Nanocages through Two Different Switches for Encapsulation of Cargo Molecules,” ACS Nano 14 (2020): 17080–17090. 33197176 10.1021/acsnano.0c06670

[39] B. Nguyen and N. H. Tolia , “Protein‐Based Antigen Presentation Platforms for Nanoparticle Vaccines,” Npj Vaccines 6 (2021): 70. 33986287 10.1038/s41541-021-00330-7 PMC8119681

[40] W. Jiang , Z. Wu , Z. Gao , et al., “Artificial Cells: Past, Present and Future,” ACS Nano 16 (2022): 15705–15733. 36226996 10.1021/acsnano.2c06104

[41] D. G. Gibson , J. I. Glass , C. Lartigue , et al., “Creation of a Bacterial Cell Controlled by a Chemically Synthesized Genome,” Science 329 (2010): 52–56. 20488990 10.1126/science.1190719

[42] C. A. Hutchison 3rd , R. Y. Chuang , V. N. Noskov , N. Assad‐Garcia , et al., “Design and Synthesis of a Minimal Bacterial Genome,” Science 351 (2016): aad6253. 27013737 10.1126/science.aad6253

[43] J. F. Pelletier , L. Sun , K. S. Wise , et al., “Genetic Requirements for Cell Division in a Genomically Minimal Cell,” Cell 184 (2021): 2430–2440. 33784496 10.1016/j.cell.2021.03.008

[44] P. van Nies , I. Westerlaken , D. Blanken , M. Salas , M. Mencía , and C. Danelon , “Self‐Replication of DNA by Its Encoded Proteins in Liposome‐Based Synthetic Cells,” Nature Communications 9 (2018): 1583. 10.1038/s41467-018-03926-1 PMC5910420 29679002

[45] Z. Chen , J. Wang , W. Sun , et al., “Synthetic Beta Cells for Fusion‐Mediated Dynamic Insulin Secretion,” Nature Chemical Biology 14 (2018): 86–93. 29083418 10.1038/nchembio.2511 PMC6053053

[46] Q. Lv , L. Cheng , Y. Lu , X. Zhang , et al., “Thermosensitive Exosome–Liposome Hybrid Nanoparticle‐Mediated Chemoimmunotherapy for Improved Treatment of Metastatic Peritoneal Cancer,” Advanced Science 7 (2020): 2000515. 32999828 10.1002/advs.202000515 PMC7509655

[47] A. C. Anselmo , Y. Gokarn , and S. Mitragotri , “Non‐Invasive Delivery Strategies for Biologics,” Nature Reviews Drug Discovery 18 (2019): 19–40. 30498202 10.1038/nrd.2018.183

[48] G. C. Terstappen , A. H. Meyer , R. D. Bell , and W. Zhang , “Strategies for Delivering Therapeutics across the Blood–Brain Barrier,” Nature Reviews Drug Discovery 20 (2021): 362–383. 33649582 10.1038/s41573-021-00139-y

[49] A. M. Vargason , A. C. Anselmo , and S. Mitragotri , “The Evolution of Commercial Drug Delivery Technologies,” Nature Biomedical Engineering 5 (2021): 951–967. 10.1038/s41551-021-00698-w 33795852

[50] X. Zhang , J. Wang , Z. Chen , et al., “Engineering PD‐1‐Presenting Platelets for Cancer Immunotherapy,” Nano Letters 18 (2018): 5716–5725. 30063143 10.1021/acs.nanolett.8b02321

[51] X. Zhang , Y. Kang , J. Wang , et al., “Engineered PD‐L1‐Expressing Platelets Reverse New‐Onset Type 1 Diabetes,” Advanced Materials 32 (2020): e1907692. 32449212 10.1002/adma.201907692

[52] C. R. Gurbatri , I. Lia , R. Vincent , et al., “Engineered Probiotics for Local Tumor Delivery of Checkpoint Blockade Nanobodies,” Science Translational Medicine 12 (2020): e1907692. 10.1126/scitranslmed.aax0876 PMC7685004 32051224

[53] S. Chowdhury , S. Castro , C. Coker , T. E. Hinchliffe , N. Arpaia , and T. Danino , “Programmable Bacteria Induce Durable Tumor Regression and Systemic Antitumor Immunity,” Nature Medicine 25 (2019): 1057–1063. 10.1038/s41591-019-0498-z PMC6688650 31270504

[54] M. Xie , H. Ye , H. Wang , et al., “β‐cell–Mimetic Designer Cells Provide Closed‐Loop Glycemic Control,” Science 354 (2016): 1296–1301. 27940875 10.1126/science.aaf4006

[55] G. M. Allen , N. W. Frankel , N. R. Reddy , et al., “Synthetic Cytokine Circuits That Drive T Cells into Immune‐Excluded Tumors,” Science 378 (2022): eaba1624. 36520915 10.1126/science.aba1624 PMC9970000

[56] J. Zhou , M. Li , Q. Chen , et al., “Programmable Probiotics Modulate Inflammation and Gut Microbiota for Inflammatory Bowel Disease Treatment after Effective Oral Delivery,” Nature Communications 13 (2022): 3432. 10.1038/s41467-022-31171-0 PMC9198027 35701435

[57] C. Yang , M. Cui , Y. Zhang , et al., “Upconversion Optogenetic Micro‐Nanosystem Optically Controls the Secretion of Light‐Responsive Bacteria for Systemic Immunity Regulation,” Communications Biology 3 (2020): 561. 33037315 10.1038/s42003-020-01287-4 PMC7547716

[58] Y. Chen , M. Du , Z. Yuan , Z. Chen , and F. Yan , “Spatiotemporal Control of Engineered Bacteria to Express Interferon‐γ by Focused Ultrasound for Tumor Immunotherapy,” Nature Communications 13 (2022): 4468. 10.1038/s41467-022-31932-x PMC9345953 35918309

[59] X. Ma , X. Liang , Y. Li , et al., “Modular‐Designed Engineered Bacteria for Precision Tumor Immunotherapy via Spatiotemporal Manipulation by Magnetic Field,” Nature Communications 14 (2023): 1606. 10.1038/s41467-023-37225-1 PMC10036336 36959204

[60] K. Krawczyk , S. Xue , P. Buchmann , et al., “Electrogenetic Cellular Insulin Release for Real‐Time Glycemic Control in Type 1 Diabetic Mice,” Science 368 (2020): 993–1001. 32467389 10.1126/science.aau7187

[61] J. Zhu , Y. Ke , Q. Liu , et al., “Engineered Lactococcus Lactis Secreting Flt3L and OX40 Ligand for in Situ Vaccination‐Based Cancer Immunotherapy,” Nature Communications 13 (2022): 7466. 10.1038/s41467-022-35130-7 PMC9719518 36463242

[62] Y. Yue , J. Xu , Y. Li , et al., “Antigen‐bearing Outer Membrane Vesicles as Tumour Vaccines Produced in Situ by Ingested Genetically Engineered Bacteria,” Nature Biomedical Engineering 6 (2022): 898–909. 10.1038/s41551-022-00886-2 35501399

[63] W. Shi , X. Yang , S. Xie , et al., “A New PD‐1‐Specific Nanobody Enhances the Antitumor Activity of T‐Cells in Synergy with Dendritic Cell Vaccine,” Cancer Letters 522 (2021): 184–197. 34562519 10.1016/j.canlet.2021.09.028

[64] M. H. Linde , A. C. Fan , T. Köhnke , et al., “Reprogramming Cancer Into Antigen‐Presenting Cells as a Novel Immunotherapy,” Cancer Discovery 13 (2023): 1164–1185. 36856575 10.1158/2159-8290.CD-21-0502

[65] A. V. R. Kornepati , R. K. Vadlamudi , and T. J. Curiel , “Programmed Death Ligand 1 Signals in Cancer Cells,” Nature Reviews Cancer 22 (2022): 174–189. 35031777 10.1038/s41568-021-00431-4 PMC9989967

[66] K. M. Hargadon , C. E. Johnson , and C. J. Williams , “Immune Checkpoint Blockade Therapy for Cancer: An Overview of FDA‐Approved Immune Checkpoint Inhibitors,” International Immunopharmacology 62 (2018): 29–39. 29990692 10.1016/j.intimp.2018.06.001

[67] L. Zhou , M. Zou , Y. Xu , P. Lin , C. Lei , and X. Xia , “Nano Drug Delivery System for Tumor Immunotherapy: Next‐Generation Therapeutics,” Frontiers in Oncology 12 (2022): 864301. 35664731 10.3389/fonc.2022.864301 PMC9160744

[68] S. Li , Z. Lu , S. Wu , et al., “The Dynamic Role of Platelets in Cancer Progression and Their Therapeutic Implications,” Nature Reviews Cancer 24 (2024): 72–87. 38040850 10.1038/s41568-023-00639-6

[69] C. Wang , W. Sun , Y. Ye , Q. Hu , H. N. Bomba , and Z. Gu , “In Situ Activation of Platelets With Checkpoint Inhibitors for Post‐Surgical Cancer Immunotherapy,” Nature Biomedical Engineering 1 (2017): 0011.

[70] Q. Hu , H. Li , E. Archibong , et al., “Inhibition of Post‐Surgery Tumour Recurrence via a Hydrogel Releasing CAR‐T Cells and Anti‐PDL1‐Conjugated Platelets,” Nature Biomedical Engineering 5 (2021): 1038–1047. 10.1038/s41551-021-00712-1 PMC9102991 33903744

[71] J. Fang , N. Ding , X. Guo , et al., “αPD‐1‐mesoCAR‐T Cells Partially Inhibit the Growth of Advanced/Refractory Ovarian Cancer in a Patient along With Daily Apatinib,” Journal for ImmunoTherapy of Cancer 9 (2021): e001162. 33589520 10.1136/jitc-2020-001162 PMC7887368

[72] M. Mansouri and M. Fussenegger , “Therapeutic Cell Engineering: Designing Programmable Synthetic Genetic Circuits in Mammalian Cells,” Protein Cell 13 (2022): 476–489. 34586617 10.1007/s13238-021-00876-1 PMC9226217

[73] M. Xie , V. Haellman , and M. Fussenegger , “Synthetic Biology — Application‐Oriented Cell Engineering,” Current Opinion in Biotechnology 40 (2016): 139–148. 27135809 10.1016/j.copbio.2016.04.005

[74] K. Rössger , G. Charpin‐El‐Hamri , and M. Fussenegger , “A Closed‐Loop Synthetic Gene Circuit for the Treatment of Diet‐Induced Obesity in Mice,” Nature Communications 4 (2013): 2825. 10.1038/ncomms3825 PMC3868331 24281397

[75] Y. Liu , P. Bai , A. K. Woischnig , et al., “Immunomimetic Designer Cells Protect Mice From MRSA Infection,” Cell 174 (2018): 259–270. 29937224 10.1016/j.cell.2018.05.039 PMC6057273

[76] J. Li , N. Li , J. Wei , et al., “Genetically Engineered Mesenchymal Stem Cells with Dopamine Synthesis for Parkinson's Disease in Animal Models,” Npj Parkinson's Disease 8 (2022): 175. 10.1038/s41531-022-00440-6 PMC9780305 36550118

[77] H. Wang , C. N. Alarcón , B. Liu , et al., “Genetically Engineered and Enucleated Human Mesenchymal Stromal Cells for the Targeted Delivery of Therapeutics to Diseased Tissue,” Nature Biomedical Engineering 6 (2022): 882–897. 10.1038/s41551-021-00815-9 PMC9207157 34931077

[78] T. Lan , L. Chen , and X. Wei , “Inflammatory Cytokines in Cancer: Comprehensive Understanding and Clinical Progress in Gene Therapy,” Cells 10 (2021): 100. 33429846 10.3390/cells10010100 PMC7827947

[79] Z. Zhang , L. Miao , Z. Ren , F. Tang , and Y. Li , “Gene‐Edited Interleukin CAR‐T Cells Therapy in the Treatment of Malignancies: Present and Future,” Frontiers in Immunology 12 (2021): 718686. 34386015 10.3389/fimmu.2021.718686 PMC8353254

[80] M. Bell and S. Gottschalk , “Engineered Cytokine Signaling to Improve CAR T Cell Effector Function,” Frontiers in Immunology 12 (2021): 684642. 34177932 10.3389/fimmu.2021.684642 PMC8220823

[81] X. Ma , P. Shou , C. Smith , et al., “Interleukin‐23 Engineering Improves CAR T Cell Function in Solid Tumors,” Nature Biotechnology 38 (2020): 448–459. 10.1038/s41587-019-0398-2 PMC7466194 32015548

[82] Y. Zhao , J. Chen , M. Andreatta , et al., “IL‐10‐Expressing CAR T Cells Resist Dysfunction and Mediate Durable Clearance of Solid Tumors and Metastases,” Nature Biotechnology 42 (2024): 1693–1704. 10.1038/s41587-023-02060-8 38168996

[83] S. Kaczanowska , D. W. Beury , V. Gopalan , et al., “Genetically Engineered Myeloid Cells Rebalance the Core Immune Suppression Program in Metastasis,” Cell 184 (2021): 2033–2052. 33765443 10.1016/j.cell.2021.02.048 PMC8344805

[84] C. Jin , J. Ma , M. Ramachandran , D. Yu , and M. Essand , “Car T Cells Expressing a Bacterial Virulence Factor Trigger Potent Bystander Antitumour Responses in Solid Cancers,” Nature Biomedical Engineering 6 (2022): 830–841. 10.1038/s41551-022-00875-5 PMC9288934 35379957

[85] A. Ghasemi , A. Martinez‐Usatorre , L. Li , et al., “Cytokine‐Armed Dendritic Cell Progenitors for Antigen‐Agnostic Cancer Immunotherapy,” Nature Cancer 5 (2024): 240–261. 37996514 10.1038/s43018-023-00668-y PMC10899110

[86] K. C. Valkenburg , A. E. de Groot , and K. J. Pienta , “Targeting the Tumour Stroma to Improve Cancer Therapy,” Nature Reviews Clinical Oncology 15 (2018): 366–381. 10.1038/s41571-018-0007-1 PMC5960434 29651130

[87] Y. He , T. Liu , S. Dai , Z. Xu , L. Wang , and F. Luo , “Tumor‐Associated Extracellular Matrix: How to Be a Potential Aide to Anti‐Tumor Immunotherapy?,” Frontiers in Cell and Developmental Biology 9 (2021): 739161. 34733848 10.3389/fcell.2021.739161 PMC8558531

[88] Y. Zhao , Y. Dong , S. Yang , et al., “Bioorthogonal Equipping CAR‐T Cells With Hyaluronidase and Checkpoint Blocking Antibody for Enhanced Solid Tumor Immunotherapy,” ACS Central Science 8 (2022): 603–614. 35647274 10.1021/acscentsci.2c00163 PMC9136969

[89] Z. Xiao , L. Todd , L. Huang , et al., “Desmoplastic Stroma Restricts T Cell Extravasation and Mediates Immune Exclusion and Immunosuppression in Solid Tumors,” Nature Communications 14 (2023): 5110. 10.1038/s41467-023-40850-5 PMC10444764 37607999

[90] J. Wang , Y. Dong , P. Ma , et al., “Intelligent Micro‐/Nanorobots for Cancer Theragnostic,” Advanced Materials 34 (2022): e2201051. 35385160 10.1002/adma.202201051

[91] J. Li , B. Esteban‐Fernández de Ávila , W. Gao , L. Zhang , and J. Wang , “Micro/Nanorobots for Biomedicine: Delivery, Surgery, Sensing, and Detoxification,” Science Robotics 2 (2017): eaam6431. 31552379 10.1126/scirobotics.aam6431 PMC6759331

[92] W. Hu , Q. Li , B. Li , K. Ma , C. Zhang , and X. Fu , “Optogenetics Sheds New Light on Tissue Engineering and Regenerative Medicine,” Biomaterials 227 (2020): 119546. 31655444 10.1016/j.biomaterials.2019.119546

[93] Z. Zhao , Q. Saiding , Z. Cai , M. Cai , and W. Cui , “Ultrasound Technology and Biomaterials for Precise Drug Therapy,” Materials Today 63 (2023): 210–238.

[94] M. Christiansen , W. Hornslien , and S. Schurle , “A Possible Inductive Mechanism for Magnetogenetics,” Biorxiv 20200716207126 (2020).

[95] S. A. Stanley , L. Kelly , K. N. Latcha , et al., “Bidirectional Electromagnetic Control of the Hypothalamus Regulates Feeding and Metabolism,” Nature 531 (2016): 647–650. 27007848 10.1038/nature17183 PMC4894494

[96] B. Hasannejad‐Asl , F. Pooresmaeil , S. Takamoli , M. Dabiri , and A. Bolhassani , “Cell‐Penetrating Peptide: A Potent Delivery System in Vaccine Development,” Frontiers in Pharmacology 13 (2022): 1072685. 36425579 10.3389/fphar.2022.1072685 PMC9679422

[97] S. Jhunjhunwala , C. Hammer , and L. Delamarre , “Antigen Presentation in Cancer: Insights into Tumour Immunogenicity and Immune Evasion,” Nature Reviews Cancer 21 (2021): 298–312. 33750922 10.1038/s41568-021-00339-z

[98] R. Zhang , X. Peng , G. Duan , et al., “An Engineered Lactococcus Lactis Strain Exerts Significant Immune Responses through Efficient Expression and Delivery of Helicobacter Pylori Lpp20 Antigen,” Biotechnology Letters 38 (2016): 2169–2175. 27646988 10.1007/s10529-016-2209-x

[99] V. Chamcha , A. Jones , B. R. Quigley , J. R. Scott , and R. R. Amara , “Oral Immunization With a Recombinant Lactococcus Lactis –Expressing HIV‐1 Antigen on Group A Streptococcus Pilus Induces Strong Mucosal Immunity in the Gut,” Journal of Immunology 195 (2015): 5025–5034. 10.4049/jimmunol.1501243 PMC4637245 26482408

[100] M. Super , E. J. Doherty , M. J. Cartwright , et al., “Biomaterial Vaccines Capturing Pathogen‐Associated Molecular Patterns Protect against Bacterial Infections and Septic Shock,” Nature Biomedical Engineering 6 (2022): 8–18. 10.1038/s41551-021-00756-3 34239117

[101] Y. E. Chen , D. Bousbaine , A. Veinbachs , et al., “Engineered Skin Bacteria Induce Antitumor T Cell Responses against Melanoma,” Science 380 (2023): 203–210. 37053311 10.1126/science.abp9563 PMC12356174

[102] R. L. Vincent , C. R. Gurbatri , F. Li , et al., “Probiotic‐Guided CAR‐T Cells for Solid Tumor Targeting,” Science 382 (2023): 211–218. 37824640 10.1126/science.add7034 PMC10915968

[103] A. Harari , M. Graciotti , M. Bassani‐Sternberg , and L. E. Kandalaft , “Antitumour Dendritic Cell Vaccination in a Priming and Boosting Approach,” Nature Reviews Drug Discovery 19 (2020): 635–652. 32764681 10.1038/s41573-020-0074-8

[104] M. Collin and V. Bigley , “Human Dendritic Cell Subsets: An Update,” Immunology 154 (2018): 3–20. 29313948 10.1111/imm.12888 PMC5904714

[105] R. L. Prue , F. Vari , K. J. Radford , et al., “A Phase I Clinical Trial of CD1c (BDCA‐1)+ Dendritic Cells Pulsed with HLA‐A*0201 Peptides for Immunotherapy of Metastatic Hormone Refractory Prostate Cancer,” Journal of Immunotherapy 38 (2015): 71–76. 25658616 10.1097/CJI.0000000000000063

[106] H. Westdorp , J. H. A. Creemers , I. M. van Oort , et al., “Blood‐Derived Dendritic Cell Vaccinations Induce Immune Responses That Correlate With Clinical Outcome in Patients With Chemo‐Naive Castration‐Resistant Prostate Cancer,” Journal for ImmunoTherapy of Cancer 7 (2019): 302. 31727154 10.1186/s40425-019-0787-6 PMC6854814

[107] J. L. Hsu , C. E. Bryant , M. S. Papadimitrious , et al., “A Blood Dendritic Cell Vaccine for Acute Myeloid Leukemia Expands Anti‐Tumor T Cell Responses at Remission,” Oncoimmunology 7 (2018): e1419114. 29632738 10.1080/2162402X.2017.1419114 PMC5889209

[108] V. Koucký , J. Bouček , and A. Fialová , “Immunology of Plasmacytoid Dendritic Cells in Solid Tumors: A Brief Review,” Cancers 11 (2019): 470. 30987228 10.3390/cancers11040470 PMC6520684

[109] S. Lee , S. Kivimäe , A. Dolor , and F. C. Szoka , “Macrophage‐Based Cell Therapies: The Long and Winding Road,” Journal of Controlled Release 240 (2016): 527–540. 27422609 10.1016/j.jconrel.2016.07.018 PMC5064880

[110] O. Zimmermannova , A. G. Ferreira , E. Ascic , et al., “Restoring Tumor Immunogenicity With Dendritic Cell Reprogramming,” Science Immunology 8 (2023): eadd4817. 37418548 10.1126/sciimmunol.add4817 PMC7614848

[111] E. A. Scott , N. B. Karabin , and P. Augsornworawat , “Overcoming Immune Dysregulation With Immunoengineered Nano‐Biomaterials,” Annual Review of Biomedical Engineering 19 (2017): 57–84. 10.1146/annurev-bioeng-071516-044603 28226216

[112] M. S. Goldberg , “Improving Cancer Immunotherapy through Nanotechnology,” Nature Reviews Cancer 19 (2019): 587–602. 31492927 10.1038/s41568-019-0186-9

[113] E. P. Stater , A. Y. Sonay , C. Hart , and J. Grimm , “The Ancillary Effects of Nanoparticles and Their Implications for Nanomedicine,” Nature Nanotechnology 16 (2021): 1180–1194. 10.1038/s41565-021-01017-9 PMC9031277 34759355

[114] P. J. Gawne , M. Ferreira , M. Papaluca , J. Grimm , and P. Decuzzi , “New Opportunities and Old Challenges in the Clinical Translation of Nanotheranostics,” Nature Reviews Materials 8 (2023): 783–798. 10.1038/s41578-023-00581-x PMC11251001 39022623

[115] B. B. Mendes , J. Conniot , A. Avital , et al., “Nanodelivery of Nucleic Acids,” Nature Reviews Methods Primers 2 (2022): 24. 10.1038/s43586-022-00104-y PMC9038125 35480987

[116] G. Liu , M. Zhu , X. Zhao , and G. Nie , “Nanotechnology‐Empowered Vaccine Delivery for Enhancing CD8+ T Cells‐Mediated Cellular Immunity,” Advanced Drug Delivery Reviews 176 (2021): 113889. 34364931 10.1016/j.addr.2021.113889

[117] A. S. Cheung , D. K. Y. Zhang , S. T. Koshy , and D. J. Mooney , “Scaffolds That Mimic Antigen‐Presenting Cells Enable Ex Vivo Expansion of Primary T Cells,” Nature Biotechnology 36 (2018): 160–169. 10.1038/nbt.4047 PMC5801009 29334370

[118] P. Agarwalla , E. A. Ogunnaike , S. Ahn , et al., “Bioinstructive Implantable Scaffolds for Rapid in Vivo Manufacture and Release of CAR‐T Cells,” Nature Biotechnology 40 (2022): 1250–1258. 10.1038/s41587-022-01245-x PMC9376243 35332339

[119] C. Chen , W. Jing , Y. Chen , et al., “Intracavity Generation of Glioma Stem Cell–Specific Car Macrophages Primes Locoregional Immunity for Postoperative Glioblastoma Therapy,” Science Translational Medicine 14 (2022): eabn1128. 35921473 10.1126/scitranslmed.abn1128

[120] S. Ling , S. Yang , X. Hu ,, et al., “Lentiviral Delivery of Co‐Packaged Cas9 MRNA and a VEGFA‐Targeting Guide RNA Prevents Wet Age‐Related Macular Degeneration in Mice,” Nature Biomedical Engineering 5 (2021): 144–156. 10.1038/s41551-020-00656-y 33398131

[121] L. Breda , T. E. Papp , M. P. Triebwasser , et al., “In Vivo Hematopoietic Stem Cell Modification by MRNA Delivery,” Science 381 (2023): 436–443. 37499029 10.1126/science.ade6967 PMC10567133

[122] X. Tang , Y. Yang , M. Zheng , et al., “Magnetic–Acoustic Sequentially Actuated CAR T Cell Microrobots for Precision Navigation and in Situ Antitumor Immunoactivation,” Advanced Materials 35 (2023): e2211509. 36807373 10.1002/adma.202211509

[123] Y. Luo , Z. Chen , M. Sun , et al., “IL‐12 Nanochaperone‐Engineered CAR T Cell for Robust Tumor‐Immunotherapy,” Biomaterials 281 (2022): 121341. 34995901 10.1016/j.biomaterials.2021.121341

[124] M.‐H. Zhu , X.‐D. Zhu , M. Long , et al., “Metal‐Coordinated Adsorption of Nanoparticles to Macrophages for Targeted Cancer Therapy,” Advanced Functional Materials 33 (2023): 2214842.

[125] Y. Dai , X. Bai , L. Jia , et al., “Precise Control of Customized Macrophage Cell Robot for Targeted Therapy of Solid Tumors With Minimal Invasion,” Small 17 (2021): e2103986. 34510759 10.1002/smll.202103986

[126] Y. Chang , X. Cai , R. Syahirah , et al., “CAR‐Neutrophil Mediated Delivery of Tumor‐Microenvironment Responsive Nanodrugs for Glioblastoma Chemo‐Immunotherapy,” Nature Communications 14 (2023): 2266. 10.1038/s41467-023-37872-4 PMC10119091 37080958

[127] H. Zhang , Z. Li , C. Gao , et al., “Dual‐Responsive Biohybrid Neutrobots for Active Target Delivery,” Science Robotics 6 (2021): eaaz9519. 34043546 10.1126/scirobotics.aaz9519

[128] Z. Luo , Y. Lu , Y. Shi , et al., “Neutrophil Hitchhiking for Drug Delivery to the Bone Marrow,” Nature Nanotechnology 18 (2023): 647–656. 10.1038/s41565-023-01374-7 37081080

[129] L. Zhang , B. Zhang , R. Liang , et al., “A Dual‐Biomineralized Yeast Micro‐/Nanorobot With Self‐Driving Penetration for Gastritis Therapy and Motility Recovery,” ACS Nano 17 (2023): 6410–6422. 36988613 10.1021/acsnano.2c11258

[130] F. Zhang , J. Zhuang , Z. Li , et al., “Nanoparticle‐Modified Microrobots for in Vivo Antibiotic Delivery to Treat Acute Bacterial Pneumonia,” Nature Materials 21 (2022): 1324–1332. 36138145 10.1038/s41563-022-01360-9 PMC9633541

[131] L. Wang , G. Wang , W. Mao , et al., “Bioinspired Engineering of Fusogen and Targeting Moiety Equipped Nanovesicles,” Nature Communications 14 (2023): 3366. 10.1038/s41467-023-39181-2 PMC10250350 37291242

[132] W. Fang , L. Li , Z. Lin , et al., “Engineered IL‐15/IL‐15R α‐Expressing Cellular Vesicles Promote T Cell Anti‐Tumor Immunity,” Extracellular Vesicle 2 (2023): 100021.

[133] T. Fang , B. Li , M. Li , et al., “Engineered Cell Membrane Vesicles Expressing CD40 Alleviate System Lupus Nephritis by Intervening B Cell Activation,” Small Methods 7 (2023): e2200925. 36605001 10.1002/smtd.202200925

[134] Y. Yu , Q. Cheng , X. Ji , et al., “Engineered Drug‐Loaded Cellular Membrane Nanovesicles for Efficient Treatment of Postsurgical Cancer Recurrence and Metastasis,” Science Advances 8 (2022): eadd3599. 36490349 10.1126/sciadv.add3599 PMC9733928

[135] X. Shi , Q. Cheng , T. Hou , et al., “Genetically Engineered Cell‐Derived Nanoparticles for Targeted Breast Cancer Immunotherapy,” Molecular Therapy 28 (2020): 536. 31843452 10.1016/j.ymthe.2019.11.020 PMC7001084

[136] Q. Cheng , Z. Dai , G. Smbatyan , et al., “Eliciting Anti‐Cancer Immunity by Genetically Engineered Multifunctional Exosomes,” Molecular Therapy 30 (2022): 3066–3077. 35746867 10.1016/j.ymthe.2022.06.013 PMC9481992

[137] R. Bhatta , J. Han , Y. Liu , et al., “Metabolic Tagging of Extracellular Vesicles and Development of Enhanced Extracellular Vesicle Based Cancer Vaccines,” Nature Communications 14 (2023): 8047. 10.1038/s41467-023-43914-8 PMC10697976 38052869

[138] K. Cheng , R. Zhao , Y. Li , et al., “Bioengineered Bacteria‐Derived Outer Membrane Vesicles as a Versatile Antigen Display Platform for Tumor Vaccination via Plug‐and‐Display Technology,” Nature Communications 12 (2021): 2041. 10.1038/s41467-021-22308-8 PMC8024398 33824314

[139] Y. Li , X. Ma , Y. Yue , et al., “Rapid Surface Display of mRNA Antigens by Bacteria‐Derived Outer Membrane Vesicles for a Personalized Tumor Vaccine,” Advanced Materials 34 (2022): e2109984. 35315546 10.1002/adma.202109984

[140] Y. Li , R. Zhao , K. Cheng , et al., “Bacterial Outer Membrane Vesicles Presenting Programmed Death 1 for Improved Cancer Immunotherapy via Immune Activation and Checkpoint Inhibition,” ACS Nano 14 (2020): 16698–16711. 33232124 10.1021/acsnano.0c03776

[141] S. Li , Q. Jiang , S. Liu , et al., “A DNA Nanorobot Functions as a Cancer Therapeutic in Response to a Molecular Trigger in Vivo,” Nature Biotechnology 36 (2018): 258–264. 10.1038/nbt.4071 29431737

[142] W. Tang , T. Tong , H. Wang , et al., “A DNA Origami‐Based Gene Editing System for Efficient Gene Therapy in Vivo,” Angewandte Chemie International Edition 62 (2023): e202315093. 37906116 10.1002/anie.202315093

[143] Z. Xu , Y. Dong , N. Ma , et al., “Confinement in Dual‐Chain‐Locked DNA Origami Nanocages Programs Marker‐Responsive Delivery of CRISPR/Cas9 Ribonucleoproteins,” Journal of the American Chemical Society 145 (2023): 26557–26568. 38039555 10.1021/jacs.3c04074

[144] Z. Wang , S. Zhang , R. Zhang , et al., “Bioengineered Dual‐Targeting Protein Nanocage for Stereoscopical Loading of Synergistic Hydrophilic/Hydrophobic Drugs to Enhance Anticancer Efficacy,” Advanced Functional Materials 31 (2021): 2102004.

[145] H. Huang , S. Yuan , Z. Ma , et al., “Genetic Recombination of Poly( L‐Lysine) Functionalized Apoferritin Nanocages That Resemble Viral Capsid Nanometer‐Sized Platforms for Gene Therapy,” Biomaterials Science 8 (2020): 1759–1770. 32010909 10.1039/c9bm01822k

[146] Y. Wu , H. Xie , Y. Li , et al., “Nitric Oxide‐Loaded Bioinspired Lipoprotein Normalizes Tumor Vessels to Improve Intratumor Delivery and Chemotherapy of Albumin‐Bound Paclitaxel Nanoparticles,” Nano Letters 23 (2023): 939–947. 36701555 10.1021/acs.nanolett.2c04312

[147] Y. Zhai , J. Wang , T. Lang , et al., “T Lymphocyte Membrane‐Decorated Epigenetic Nanoinducer of Interferons for Cancer Immunotherapy,” Nature Nanotechnology 16 (2021): 1271–1280. 10.1038/s41565-021-00972-7 34580467

[148] Q. Tang , S. Sun , P. Wang , et al., “Genetically Engineering Cell Membrane‐Coated BTO Nanoparticles for MMP2‐Activated Piezocatalysis‐Immunotherapy,” Advanced Materials 35 (2023): e2300964. 36809650 10.1002/adma.202300964

[149] S. Koo , H. S. Sohn , T. H. Kim , et al., “Ceria‐Vesicle Nanohybrid Therapeutic for Modulation of Innate and Adaptive Immunity in a Collagen‐Induced Arthritis Model,” Nature Nanotechnology 18 (2023): 1502–1514. 10.1038/s41565-023-01523-y 37884660

[150] C. Simó , M. Serra‐Casablancas , A. C. Hortelao , et al., “Urease‐Powered Nanobots for Radionuclide Bladder Cancer Therapy,” Nature Nanotechnology 19 (2024): 554–564. 10.1038/s41565-023-01577-y PMC11026160 38225356

[151] M. Yan , Q. Chen , T. Liu , et al., “Site‐Selective Superassembly of Biomimetic Nanorobots Enabling Deep Penetration Into Tumor With Stiff Stroma,” Nature Communications 14 (2023): 4628. 10.1038/s41467-023-40300-2 PMC10397308 37532754

[152] N. Yin , W. Zhang , X. X. Sun , et al., “Artificial Cells Delivering Itaconic Acid Induce Anti‐Inflammatory Memory‐Like Macrophages to Reverse Acute Liver Failure and Prevent Reinjury,” Cell Reports Medicine 4 (2023): 101132. 37541252 10.1016/j.xcrm.2023.101132 PMC10439255

[153] R. Liu , W. Wang , Y. Wang , L. Zhang , and G. Chen , “The Preliminary Study on Preparation Technology of PolyHb‐SOD‐CATCA—The Effects of Different Extractants,” Current Pharmaceutical Biotechnology 24 (2023): 1928–1937. 37005550 10.2174/1389201024666230331083354

[154] Y. Ma , H. Yang , X. Zong , et al., “Artificial M2 Macrophages for Disease‐Modifying Osteoarthritis Therapeutics,” Biomaterials 274 (2021): 120865. 33991950 10.1016/j.biomaterials.2021.120865

[155] P. Xiao , J. Wang , Z. Zhao , et al., “Engineering Nanoscale Artificial Antigen‐Presenting Cells by Metabolic Dendritic Cell Labeling to Potentiate Cancer Immunotherapy,” Nano Letters 21 (2021): 2094–2103. 33622034 10.1021/acs.nanolett.0c04783

[156] D. J. Irvine , M. V. Maus , D. J. Mooney , and W. W. Wong , “The Future of Engineered Immune Cell Therapies,” Science 378 (2022): 853–858. 36423279 10.1126/science.abq6990 PMC9919886

[157] H. S. Kim , T. C. Ho , M. J. Willner , M. W. Becker , H. W. Kim , and K. W. Leong , “Dendritic Cell‐Mimicking Scaffolds for Ex Vivo T Cell Expansion,” Bioactive Materials 21 (2023): 241–252. 36157246 10.1016/j.bioactmat.2022.08.015 PMC9474324

[158] D. H. Gutmann and H. Kettenmann , “Microglia/Brain Macrophages as Central Drivers of Brain Tumor Pathobiology,” Neuron 104 (2019): 442–449. 31697921 10.1016/j.neuron.2019.08.028 PMC7288606

[159] C. Garris and M. J. Pittet , “Therapeutically Reeducating Macrophages to Treat GBM,” Nature Medicine 19 (2013): 1207–1208. 10.1038/nm.3355 24100977

[160] J. H. Sampson , M. D. Gunn , P. E. Fecci , and D. M. Ashley , “Brain Immunology and Immunotherapy in Brain Tumours,” Nature Reviews Cancer 20 (2020): 12–25. 31806885 10.1038/s41568-019-0224-7 PMC7327710

[161] H. Wang , M. C. Sobral , D. K. Y. Zhang , et al., “Metabolic Labeling and Targeted Modulation of Dendritic Cells,” Nature Materials 19 (2020): 1244–1252. 32424368 10.1038/s41563-020-0680-1 PMC7748064

[162] H. Du , J. M. Bartleson , S. Butenko , et al., “Tuning Immunity through Tissue Mechanotransduction,” Nature Reviews Immunology 23 (2023): 174–188. 10.1038/s41577-022-00761-w PMC9379893 35974148

[163] K. Lei , A. Kurum , M. Kaynak , et al., “Cancer‐Cell Stiffening via Cholesterol Depletion Enhances Adoptive T‐Cell Immunotherapy,” Nature Biomedical Engineering 5 (2021): 1411–1425. 10.1038/s41551-021-00826-6 PMC7612108 34873307

[164] W. A. Nyberg , J. Ark , A. To , et al., “An Evolved AAV Variant Enables Efficient Genetic Engineering of Murine T Cells,” Cell 186 (2023): 446–460. 36638795 10.1016/j.cell.2022.12.022 PMC10540678

[165] T. Kerzel , G. Giacca , S. Beretta , et al., “In Vivo Macrophage Engineering Reshapes the Tumor Microenvironment Leading to Eradication of Liver Metastases,” Cancer Cell 41 (2023): 1892–1910. 37863068 10.1016/j.ccell.2023.09.014

[166] A. D. Nahmad , C. R. Lazzarotto , N. Zelikson , et al., “In Vivo Engineered B Cells Secrete High Titers of Broadly Neutralizing Anti‐HIV Antibodies in Mice,” Nature Biotechnology 40 (2022): 1241–1249. 10.1038/s41587-022-01328-9 PMC7613293 35681059

[167] D. Yin , S. Ling , D. Wang , et al., “Targeting Herpes Simplex Virus with CRISPR–Cas9 Cures Herpetic Stromal Keratitis in Mice,” Nature Biotechnology 39 (2021): 567–577. 10.1038/s41587-020-00781-8 PMC7611178 33432198

[168] S. Banskota , A. Raguram , S. Suh , et al., “Engineered Virus‐Like Particles for Efficient in Vivo Delivery of Therapeutic Proteins,” Cell 185 (2022): 250–265. 35021064 10.1016/j.cell.2021.12.021 PMC8809250

[169] M. An , A. Raguram , S. W. Du , et al., “Engineered Virus‐Like Particles for Transient Delivery of Prime Editor Ribonucleoprotein Complexes in Vivo,” Nature Biotechnology 185 (2024): 250–265. 10.1038/s41587-023-02078-y PMC11228131 38191664

[170] C. J. Bao , J. L. Duan , Y. Xie , et al., “Bioorthogonal Engineered Virus‐Like Nanoparticles for Efficient Gene Therapy,” Nano‐Micro Letters 15 (2023): 197. 37572220 10.1007/s40820-023-01153-y PMC10423197

[171] J. R. Hamilton , E. Chen , B. S. Perez , et al., “In Vivo Human T Cell Engineering With Enveloped Delivery Vehicles,” Nature Biotechnology 185 (2024): 250–265. 10.1038/s41587-023-02085-z PMC11236958 38212493

[172] S. A. Dilliard , Q. Cheng , and D. J. Siegwart , “On the Mechanism of Tissue‐Specific mRNA Delivery by Selective Organ Targeting Nanoparticles,” Proceedings of the National Academy of Sciences of the United States of America 118 (2021): e2109256118. 34933999 10.1073/pnas.2109256118 PMC8719871

[173] Q. Cheng , T. Wei , L. Farbiak , L. T. Johnson , S. A. Dilliard , and D. J. Siegwart , “Selective Organ Targeting (Sort) Nanoparticles for Tissue‐Specific mRNA Delivery and CRISPR–CAS Gene Editing,” Nature Nanotechnology 15 (2020): 313–320. 10.1038/s41565-020-0669-6 PMC7735425 32251383

[174] L. M. Kranz , M. Diken , H. Haas , et al., “Systemic RNA Delivery to Dendritic Cells Exploits Antiviral Defence for Cancer Immunotherapy,” Nature 534 (2016): 396–401. 27281205 10.1038/nature18300

[175] J. G. Rurik , I. Tombácz , A. Yadegari , et al., “CAR T Cells Produced in Vivo to Treat Cardiac Injury,” Science 375 (2022): 91–96. 34990237 10.1126/science.abm0594 PMC9983611

[176] M. Yu , W. Song , F. Tian , et al., “Temperature‐ and Rigidity‐Mediated Rapid Transport of Lipid Nanovesicles in Hydrogels,” Proceedings of the National Academy of Sciences of the United States of America 116 (2019): 5362–5369. 30837316 10.1073/pnas.1818924116 PMC6431219

[177] Z. Belhadj , Y. Qie , R. P. Carney , Y. Li , and G. Nie , “Current Advances in Non‐Viral Gene Delivery Systems: Liposomes versus Extracellular Vesicles,” BMEMat 1 (2023): e12018.

[178] S. Liu , Q. Cheng , T. Wei , et al., “Membrane‐Destabilizing Ionizable Phospholipids for Organ‐Selective mRNA Delivery and CRISPR–Cas Gene Editing,” Nature Materials 20 (2021): 701–710. 33542471 10.1038/s41563-020-00886-0 PMC8188687

[179] F. Ma , L. Yang , Z. Sun , J. Chen , X. Rui , Z. Glass , and Q. Xu , “Neurotransmitter‐Derived Lipidoids (NT‐lipidoids) for Enhanced Brain Delivery through Intravenous Injection,” Science Advances 6 (2020): eabb4429. 32832671 10.1126/sciadv.abb4429 PMC7439549

[180] H. Li , Y. Tong , L. Bai , L. Ye , L. Zhong , X. Duan , and Y. Zhu , “Lactoferrin Functionalized PEG‐PLGA Nanoparticles of Shikonin for Brain Targeting Therapy of Glioma,” International Journal of Biological Macromolecules 107 (2018): 204–211. 28863897 10.1016/j.ijbiomac.2017.08.155

[181] D. E. Tylawsky , H. Kiguchi , J. Vaynshteyn , et al., “P‐Selectin‐Targeted Nanocarriers Induce Active Crossing of the Blood–Brain Barrier via Caveolin‐1‐Dependent Transcytosis,” Nature Materials 22 (2023): 391–399. 36864161 10.1038/s41563-023-01481-9 PMC9981459

[182] S. Liu , X. Wang , X. Yu , et al., “Zwitterionic Phospholipidation of Cationic Polymers Facilitates Systemic mRNA Delivery to Spleen and Lymph Nodes,” Journal of the American Chemical Society 143 (2021): 21321–21330. 34878786 10.1021/jacs.1c09822 PMC9437927

[183] L. Alvarez‐Erviti , Y. Seow , H. Yin , C. Betts , S. Lakhal , and M. J. A. Wood , “Delivery of siRNA to the Mouse Brain by Systemic Injection of Targeted Exosomes,” Nature Biotechnology 29 (2011): 341–345. 10.1038/nbt.1807 21423189

[184] L. Ortega‐Pineda , A. Sunyecz , A. I. Salazar‐Puerta , et al., “Designer Extracellular Vesicles Modulate Pro‐Neuronal Cell Responses and Improve Intracranial Retention,” Advanced Healthcare Materials 11 (2022): e2100805. 35014204 10.1002/adhm.202100805 PMC9466406

[185] Z. Hosseinidoust , B. Mostaghaci , O. Yasa , B. W. Park , A. V. Singh , and M. Sitti , “Bioengineered and Biohybrid Bacteria‐Based Systems for Drug Delivery,” Advanced Drug Delivery Reviews 106 (2016): 27–44. 27641944 10.1016/j.addr.2016.09.007

[186] O. Felfoul , M. Mohammadi , S. Taherkhani , et al., “Magneto‐Aerotactic Bacteria Deliver Drug‐Containing Nanoliposomes to Tumour Hypoxic Regions,” Nature Nanotechnology 11 (2016): 941–947. 10.1038/nnano.2016.137 PMC6094936 27525475

[187] M. B. Akolpoglu , Y. Alapan , N. O. Dogan , et al., “Magnetically Steerable Bacterial Microrobots Moving in 3d Biological Matrices for Stimuli‐Responsive Cargo Delivery,” Science Advances 8 (2022): eabo6163. 35857516 10.1126/sciadv.abo6163 PMC9286503

[188] H. Xu , M. Medina‐Sánchez , M. F. Maitz , C. Werner , and O. G. Schmidt , “Sperm Micromotors for Cargo Delivery Through Flowing Blood,” ACS Nano 14 (2020): 2982–2993. 32096976 10.1021/acsnano.9b07851

[189] Q. Chen , S. Tang , Y. Li , et al., “Multifunctional Metal–Organic Framework Exoskeletons Protect Biohybrid Sperm Microrobots for Active Drug Delivery from the Surrounding Threats,” ACS Applied Materials & Interfaces Journal 13 (2021): 58382–58392. 10.1021/acsami.1c18597 34860489

[190] F. Cao , L. Jin , Y. Gao , et al., “Artificial‐Enzymes‐Armed Bifidobacterium Longum Probiotics for Alleviating Intestinal Inflammation and Microbiota Dysbiosis,” Nature Nanotechnology 18 (2023): 617–627. 10.1038/s41565-023-01346-x 36973397

[191] J. Ali , U. K. Cheang , J. D. Martindale , M. Jabbarzadeh , H. C. Fu , and M. J. Kim , “Bacteria‐Inspired Nanorobots With Flagellar Polymorphic Transformations and Bundling,” Scientific Reports 7 (2017): 14098. 29074862 10.1038/s41598-017-14457-y PMC5658443

[192] H. Xu , M. Medina‐Sánchez , V. Magdanz , L. Schwarz , F. Hebenstreit , and O. G. Schmidt , “Sperm‐Hybrid Micromotor for Targeted Drug Delivery,” ACS Nano 12 (2018): 327–337. 29202221 10.1021/acsnano.7b06398

[193] T. Gwisai , N. Mirkhani , M. G. Christiansen , T. T. Nguyen , V. Ling , and S. Schuerle , “Magnetic Torque–Driven Living Microrobots for Increased Tumor Infiltration,” Science Robotics 7 (2022): eabo0665. 36288270 10.1126/scirobotics.abo0665

[194] S. Osuka and E. G. Van Meir , “Neutrophils Traffic in Cancer Nanodrugs,” Nature Nanotechnology 12 (2017): 616–618. 10.1038/nnano.2017.82 28650438

[195] Y. Chen , K. Li , M. Jiao , et al., “Reprogrammed siTNFα/Neutrophil Cytopharmaceuticals Targeting Inflamed Joints for Rheumatoid Arthritis Therapy,” Acta Pharmaceutica Sinica B 13 (2023): 787–803. 36873164 10.1016/j.apsb.2022.08.012 PMC9978920

[196] J. Shao , M. Xuan , H. Zhang , X. Lin , Z. Wu , and Q. He , “Chemotaxis‐Guided Hybrid Neutrophil Micromotors for Targeted Drug Transport,” Angewandte Chemie International Edition 56 (2017): 12935–12939. 28816386 10.1002/anie.201706570

[197] C. Martin , P. C. Burdon , G. Bridger , J. C. Gutierrez‐Ramos , T. J. Williams , and S. M. Rankin , “Chemokines Acting via CXCR2 and CXCR4 Control the Release of Neutrophils From the Bone Marrow and Their Return Following Senescence,” Immunity 19 (2003): 583–593. 14563322 10.1016/s1074-7613(03)00263-2

[198] J. Wang , M. Hossain , A. Thanabalasuriar , M. Gunzer , C. Meininger , and P. Kubes , “Visualizing the Function and Fate of Neutrophils in Sterile Injury and Repair,” Science 358 (2017): 111–116. 28983053 10.1126/science.aam9690

[199] H. Garner and K. E. de Visser , “Neutrophils Take a Round‐Trip,” Science 358 (2017): 42–43. 28983037 10.1126/science.aap8361

[200] J. Xue , Z. Zhao , L. Zhang , et al., “Neutrophil‐Mediated Anticancer Drug Delivery for Suppression of Postoperative Malignant Glioma Recurrence,” Nature Nanotechnology 12 (2017): 692–700. 10.1038/nnano.2017.54 28650441

[201] Z. Gao , N. Wang , Y. Ma , et al., “Targeting Neutrophils Potentiates Hitchhiking Delivery of Drugs and Agonists for Postsurgical Chemo‐Immunotherapy,” Nano Today 54 (2024): 102096.

[202] M. Li , S. Li , H. Zhou , et al., “Chemotaxis‐Driven Delivery of Nano‐Pathogenoids for Complete Eradication of Tumors Post‐Phototherapy,” Nature Communications 11 (2020): 1126. 10.1038/s41467-020-14963-0 PMC7048836 32111847

[203] J. Kuang , Z.‐Y. Rao , D.‐W. Zheng , et al., “Nanoparticles Hitchhike on Monocytes for Glioblastoma Treatment After Low‐Dose Radiotherapy,” ACS Nano 17 (2023): 13333–13347. 37404077 10.1021/acsnano.3c01428

[204] L. Zheng , X. Hu , H. Wu , et al., “In Vivo Monocyte/Macrophage‐Hitchhiked Intratumoral Accumulation of Nanomedicines for Enhanced Tumor Therapy,” Journal of the American Chemical Society 142 (2020): 382–391. 31801020 10.1021/jacs.9b11046

[205] F. Zhang , Z. Li , Y. Duan , et al., “Gastrointestinal Tract Drug Delivery Using Algae Motors Embedded in a Degradable Capsule,” Science Robotics 7 (2022): eabo4160. 36170380 10.1126/scirobotics.abo4160 PMC9884493

[206] Z. Chen , H. Pan , Y. Luo , et al., “Nanoengineered CAR‐T Biohybrids for Solid Tumor Immunotherapy With Microenvironment Photothermal‐Remodeling Strategy,” Small 17 (2021): e2007494. 33711191 10.1002/smll.202007494

[207] L. Tang , Y. Zheng , M. B. Melo , et al., “Enhancing T Cell Therapy through TCR‐Signaling‐Responsive Nanoparticle Drug Delivery,” Nature Biotechnology 36 (2018): 707–716. 10.1038/nbt.4181 PMC6078803 29985479

[208] C. W. Shields , M. A. Evans , L. L. Wang , et al., “Cellular Backpacks for Macrophage Immunotherapy,” Science Advances 6 (2020): eaaz6579. 32494680 10.1126/sciadv.aaz6579 PMC7190308

[209] C. X. Li , Y. Zhang , X. Dong , et al., “Artificially Reprogrammed Macrophages as Tumor‐Tropic Immunosuppression‐Resistant Biologics to Realize Therapeutics Production and Immune Activation,” Advanced Materials 31 (2019): e1807211. 30803083 10.1002/adma.201807211

[210] F. Zhang , Z. Li , L. Yin , et al., “ACE2 Receptor‐Modified Algae‐Based Microrobot for Removal of SARS‐CoV‐2 in Wastewater,” Journal of the American Chemical Society 143 (2021): 12194–12201. 34291944 10.1021/jacs.1c04933

[211] J. Lai , Q. F. Meng , M. Tian , et al., “A Decoy Microrobot That Removes SARS‐CoV‐2 and Its Variants in Wastewater,” Cell Reports Physical Science 3 (2022): 101061. 36158867 10.1016/j.xcrp.2022.101061 PMC9490858

[212] B. Mostaghaci , O. Yasa , J. Zhuang , and M. Sitti , “Bioadhesive Bacterial Microswimmers for Targeted Drug Delivery in the Urinary and Gastrointestinal Tracts,” Advanced Science 4 (2017): 1700058. 28638787 10.1002/advs.201700058 PMC5473323

[213] W. Yang , Y. Bai , Y. Xiong , et al., “Potentiating the Antitumour Response of CD8+ T Cells by Modulating Cholesterol Metabolism,” Nature 531 (2016): 651–655. 26982734 10.1038/nature17412 PMC4851431

[214] S. A. Lim , W. Su , N. M. Chapman , and H. Chi , “Lipid Metabolism in T Cell Signaling and Function,” Nature Chemical Biology 18 (2022): 470–481. 35484263 10.1038/s41589-022-01017-3 PMC11103273

[215] M. Hao , S. Hou , W. Li , et al., “Combination of Metabolic Intervention and T Cell Therapy Enhances Solid Tumor Immunotherapy,” Science Translational Medicine 12 (2020): eaaz6667. 33239389 10.1126/scitranslmed.aaz6667

[216] C. Shi , Q. Zhang , Y. Yao , et al., “Targeting the Activity of T Cells by Membrane Surface Redox Regulation for Cancer Theranostics,” Nature Nanotechnology 18 (2023): 86–97. 10.1038/s41565-022-01261-7 36536041

[217] Q. Yang , R. Liu , Q. Yu , Y. Bi , and G. Liu , “Metabolic Regulation of Inflammasomes in Inflammation,” Immunology 157 (2019): 95–109. 30851192 10.1111/imm.13056 PMC6526672

[218] D. W. Zheng , L. Xu , C. X. Li , et al., “Photo‐Powered Artificial Organelles for ATP Generation and Life‐Sustainment,” Advanced Materials 30 (2018): e1805038. 30378187 10.1002/adma.201805038

[219] J. M. Diaz , C. M. Hansel , B. M. Voelker , C. M. Mendes , P. F. Andeer , and T. Zhang , “Widespread Production of Extracellular Superoxide by Heterotrophic Bacteria,” Science 340 (2013): 1223–1226. 23641059 10.1126/science.1237331

[220] P. Chen , X. Liu , C. Gu , et al., “A Plant‐Derived Natural Photosynthetic System for Improving Cell Anabolism,” Nature 612 (2022): 546–554. 36477541 10.1038/s41586-022-05499-y PMC9750875

[221] R. Yang , J. Xu , L. Xu , et al., “Cancer Cell Membrane‐Coated Adjuvant Nanoparticles With Mannose Modification for Effective Anticancer Vaccination,” ACS Nano 12 (2018): 5121–5129. 29771487 10.1021/acsnano.7b09041

[222] O. P. Wiklander , J. Z. Nordin , A. O'Loughlin , et al., “Extracellular Vesicle in Vivo Biodistribution Is Determined by Cell Source, Route of Administration and Targeting,” Journal of Extracellular Vesicles 4 (2015): 26316. 25899407 10.3402/jev.v4.26316 PMC4405624

[223] D. Ingato , J. U. Lee , S. J. Sim , and Y. J. Kwon , “Good Things Come in Small Packages: Overcoming Challenges to Harness Extracellular Vesicles for Therapeutic Delivery,” Journal of Controlled Release 241 (2016): 174–185. 27667180 10.1016/j.jconrel.2016.09.016

[224] T. Xue , Z. Zhang , T. Fang , et al., “Cellular Vesicles Expressing PD‐1‐Blocking scFv Reinvigorate T Cell Immunity against Cancer,” Nano Research 15 (2022): 5295–5304.

[225] L. Ding , X. Zhang , P. Yu , et al., “Genetically Engineered Nanovesicles Mobilize Synergistic Antitumor Immunity by ADAR1 Silence and PDL1 Blockade,” Molecular Therapy 31 (2023): 2489–2506. 37087570 10.1016/j.ymthe.2023.04.011 PMC10422002

[226] C. Liu , X. Liu , X. Xiang , et al., “A Nanovaccine for Antigen Self‐Presentation and Immunosuppression Reversal as a Personalized Cancer Immunotherapy Strategy,” Nature Nanotechnology 17 (2022): 531–540. 10.1038/s41565-022-01098-0 35410368

[227] K. Wang , X. Zhang , H. Ye , et al., “Biomimetic Nanovaccine‐Mediated Multivalent IL‐15 Self‐Transpresentation (MIST) for Potent and Safe Cancer Immunotherapy,” Nature Communications 14 (2023): 6748. 10.1038/s41467-023-42155-z PMC10598200 37875481

[228] L. Rao , S. Xia , W. Xu , et al., “Decoy Nanoparticles Protect against COVID‐19 by Concurrently Adsorbing Viruses and Inflammatory Cytokines,” Proceedings of the National Academy of Sciences of the United States of America 117 (2020): 27141–27147. 33024017 10.1073/pnas.2014352117 PMC7959535

[229] L. Cheng and A. F. Hill , “Therapeutically Harnessing Extracellular Vesicles,” Nature Reviews Drug Discovery 21 (2022): 379–399. 35236964 10.1038/s41573-022-00410-w

[230] H. Shao , H. Im , C. M. Castro , X. Breakefield , R. Weissleder , and H. Lee , “New Technologies for Analysis of Extracellular Vesicles,” Chemical Reviews 118 (2018): 1917–1950. 29384376 10.1021/acs.chemrev.7b00534 PMC6029891

[231] L. Wang , D. Wang , Z. Ye , and J. Xu , “Engineering Extracellular Vesicles as Delivery Systems in Therapeutic Applications,” Advanced Science 10 (2023): e2300552. 37080941 10.1002/advs.202300552 PMC10265081

[232] P. E. Martinez de Castilla , L. Tong , C. Huang , et al., “Extracellular Vesicles as a Drug Delivery System: A Systematic Review of Preclinical Studies,” Advanced Drug Delivery Reviews 175 (2021): 113801. 34015418 10.1016/j.addr.2021.05.011

[233] X. Zhang , H. Cui , W. Zhang , Z. Li , and J. Gao , “Engineered Tumor Cell‐Derived Vaccines against Cancer: The Art of Combating Poison With Poison,” Bioactive Materials 22 (2023): 491–517. 36330160 10.1016/j.bioactmat.2022.10.016 PMC9619151

[234] M. Morishita , Y. Takahashi , A. Matsumoto , M. Nishikawa , and Y. Takakura , “Exosome‐Based Tumor Antigens–Adjuvant Co‐Delivery Utilizing Genetically Engineered Tumor Cell‐Derived Exosomes with Immunostimulatory CpG DNA,” Biomaterials 111 (2016): 55–65. 27723556 10.1016/j.biomaterials.2016.09.031

[235] S. H. Kim , N. Bianco , R. Menon , E. R. Lechman , W. J. Shufesky , A. E. Morelli , and P. D. Robbins , “Exosomes Derived From Genetically Modified DC Expressing FasL Are Anti‐inflammatory and Immunosuppressive,” Molecular Therapy 13 (2006): 289–300. 16275099 10.1016/j.ymthe.2005.09.015

[236] C. Schwechheimer and M. J. Kuehn , “Outer‐Membrane Vesicles From Gram‐Negative Bacteria: Biogenesis and Functions,” Nature Reviews Microbiology 13 (2015): 605–619. 26373371 10.1038/nrmicro3525 PMC5308417

[237] C. Irene , L. Fantappiè , E. Caproni , et al., “Bacterial Outer Membrane Vesicles Engineered with Lipidated Antigens as a Platform for Staphylococcus Aureus Vaccine,” Proceedings of the National Academy of Sciences of the United States of America 116 (2019): 21780–21788. 31591215 10.1073/pnas.1905112116 PMC6815149

[238] K. Kuipers , M. H. Daleke‐Schermerhorn , W. S. Jong , et al., “Salmonella Outer Membrane Vesicles Displaying High Densities of Pneumococcal Antigen at the Surface Offer Protection against Colonization,” Vaccine 33 (2015): 2022–2029. 25776921 10.1016/j.vaccine.2015.03.010

[239] M. L. Salverda , S. M. Meinderts , H. J. Hamstra , et al., “Surface Display of a Borrelial Lipoprotein on Meningococcal Outer Membrane Vesicles,” Vaccine 34 (2016): 1025–1033. 26801064 10.1016/j.vaccine.2016.01.019

[240] O. Y. Kim , H. T. Park , N. T. H. Dinh , et al., “Bacterial Outer Membrane Vesicles Suppress Tumor by Interferon‐Γ‐Mediated Antitumor Response,” Nature Communications 8 (2017): 626. 10.1038/s41467-017-00729-8 PMC5606984 28931823

[241] G. Liu , N. Ma , K. Cheng , et al., “Bacteria‐Derived Nanovesicles Enhance Tumour Vaccination by Trained Immunity,” Nature Nanotechnology 19 (2024): 387–398. 10.1038/s41565-023-01553-6 38052943

[242] W. Ma , Y. Zhan , Y. Zhang , C. Mao , X. Xie , and Y. Lin , “The Biological Applications of DNA Nanomaterials: Current Challenges and Future Directions,” Signal Transduction and Targeted Therapy 6 (2021): 351. 34620843 10.1038/s41392-021-00727-9 PMC8497566

[243] J. Wang , Y. Li , and G. Nie , “Multifunctional Biomolecule Nanostructures for Cancer Therapy,” Nature Reviews Materials 6 (2021): 766–783. 34026278 10.1038/s41578-021-00315-x PMC8132739

[244] Y. Dong , C. Yao , Y. Zhu , L. Yang , D. Luo , and D. Yang , “DNA Functional Materials Assembled From Branched DNA: Design, Synthesis, and Applications,” Chemical Reviews 120 (2020): 9420. 32672036 10.1021/acs.chemrev.0c00294

[245] N. C. Seeman and H. F. Sleiman , “DNA Nanotechnology,” Nature Reviews Materials 3 (2017): 17068.

[246] P. W. Rothemund , “Folding DNA to Create Nanoscale Shapes and Patterns,” Nature 440 (2006): 297–302. 16541064 10.1038/nature04586

[247] Y. Ke , L. L. Ong , W. M. Shih , and P. Yin , “Three‐Dimensional Structures Self‐Assembled From DNA Bricks,” Science 338 (2012): 1177–1183. 23197527 10.1126/science.1227268 PMC3843647

[248] L. L. Ong , N. Hanikel , O. K. Yaghi , et al., “Programmable Self‐Assembly of Three‐Dimensional Nanostructures From 10,000 Unique Components,” Nature 552 (2017): 72–77. 29219968 10.1038/nature24648 PMC5786436

[249] S. Liu , Q. Jiang , X. Zhao , et al., “A DNA Nanodevice‐Based Vaccine for Cancer Immunotherapy,” Nature Materials 20 (2021): 421–430. 32895504 10.1038/s41563-020-0793-6

[250] Y. Li and J. A. Champion , “Self‐Assembling Nanocarriers From Engineered Proteins: Design, Functionalization, and Application for Drug Delivery,” Advanced Drug Delivery Reviews 189 (2022): 114462. 35934126 10.1016/j.addr.2022.114462

[251] P. V. Candelaria , L. S. Leoh , M. L. Penichet , and T. R. Daniels‐Wells , “Antibodies Targeting the Transferrin Receptor 1 (TfR1) as Direct Anti‐Cancer Agents,” Frontiers in Immunology 12 (2021): 607692. 33815364 10.3389/fimmu.2021.607692 PMC8010148

[252] E. J. Lee , S. J. Lee , Y.‐S. Kang , et al., “Engineered Proteinticles for Targeted Delivery of siRNA to Cancer Cells,” Advanced Functional Materials 25 (2015): 1279–1286.

[253] B. Zhang , X. Chen , G. Tang , et al., “Piezoelectric Enhanced Peroxidase‐Like Activity of Metal‐Free Sulfur Doped Graphdiyne Nanosheets for Efficient Water Pollutant Degradation and Bacterial Disinfection,” Nano Today 43 (2022): 101429.

[254] R. Wang , X. Zhang , K. Feng , et al., “Nanotechnologies Meeting Natural Sources: Engineered Lipoproteins for Precise Brain Disease Theranostics,” Asian Journal of Pharmaceutical Sciences 18 (2023): 100857. 37953874 10.1016/j.ajps.2023.100857 PMC10637878

[255] S. EL Andaloussi , I. Mäger , X. O. Breakefield , and M. J. Wood , “Extracellular Vesicles: Biology and Emerging Therapeutic Opportunities,” Nature Reviews Drug Discovery 12 (2013): 347–357. 23584393 10.1038/nrd3978

[256] Y. Wang , H. Xie , Y. Wu , et al., “Bioinspired Lipoproteins of Furoxans–Oxaliplatin Remodel Physical Barriers in Tumor to Potentiate T‐Cell Infiltration,” Advanced Materials 34 (2022): e2110614. 35092711 10.1002/adma.202110614

[257] T. Tan , H. Hu , H. Wang , et al., “Bioinspired Lipoproteins‐Mediated Photothermia Remodels Tumor Stroma to Improve Cancer Cell Accessibility of Second Nanoparticles,” Nature Communications 10 (2019): 3322. 10.1038/s41467-019-11235-4 PMC6658501 31346166

[258] R. H. Fang , W. Gao , and L. Zhang , “Targeting Drugs to Tumours Using Cell Membrane‐Coated Nanoparticles,” Nature Reviews Clinical Oncology 20 (2023): 33–48. 10.1038/s41571-022-00699-x 36307534

[259] N. Krishnan , Y. Jiang , J. Zhou , et al., “A Modular Approach to Enhancing Cell Membrane‐Coated Nanoparticle Functionality Using Genetic Engineering,” Nature Nanotechnology 19 (2024): 345–353. 10.1038/s41565-023-01533-w PMC10954421 37903891

[260] Z. Wu , Y. Chen , D. Mukasa , O. S. Pak , and W. Gao , “Medical Micro/Nanorobots in Complex Media,” Chemical Society Reviews 49 (2020): 8088–8112. 32596700 10.1039/d0cs00309c

[261] B. J. Nelson and S. Pané , “Delivering Drugs with Microrobots,” Science 382 (2023): 1120–1122. 38060660 10.1126/science.adh3073

[262] T. Li , S. Yu , B. Sun , et al., “Bioinspired Claw‐Engaged and Biolubricated Swimming Microrobots Creating Active Retention in Blood Vessels,” Science Advances 9 (2023): eadg4501. 37146139 10.1126/sciadv.adg4501 PMC10162671

[263] N. Arulkumaran , M. Singer , S. Howorka , and J. R. Burns , “Creating Complex Protocells and Prototissues Using Simple DNA Building Blocks,” Nature Communications 14 (2023): 1314. 10.1038/s41467-023-36875-5 PMC10006096 36898984

[264] C. Zhang , L. Zhang , W. Wu , et al., “Artificial Super Neutrophils for Inflammation Targeting and HClO Generation against Tumors and Infections,” Advanced Materials 31 (2019): e1901179. 30924234 10.1002/adma.201901179

[265] S. Cheng , C. Xu , Y. Jin , et al., “Artificial Mini Dendritic Cells Boost T Cell–Based Immunotherapy for Ovarian Cancer,” Advanced Science 7 (2020): 1903301. 32274314 10.1002/advs.201903301 PMC7141030

[266] Y. Jiang , N. Krishnan , J. Zhou , et al., “Engineered Cell‐Membrane‐Coated Nanoparticles Directly Present Tumor Antigens to Promote Anticancer Immunity,” Advanced Materials 32 (2020): e2001808. 32538494 10.1002/adma.202001808 PMC7669572

[267] Y. Wang and T. M. Swi Chang , “Biodegradable Nanocapsules Containing a Nanobiotechnological Complex for the In‐vitro Suppression of a Melanoma Cell Line B16F10,” Journal of Nanosciences: Current Research 01 (2016): 1000102.

[268] D. Machover , L. Rossi , J. Hamelin , et al., “Effects in Cancer Cells of the Recombinant L‐Methionine Gamma‐Lyase From Brevibacterium Aurantiacum. Encapsulation in Human Erythrocytes for Sustained L‐Methionine Elimination,” Journal of Pharmacology and Experimental Therapeutics 369 (2019): 489–502. 30940696 10.1124/jpet.119.256537

[269] F. Yaman , A. Adler , and J. Beal , “AI Challenges in Synthetic Biology Engineering.” AAAI'18/IAAI'18/EAAI'18 AAAI Press, Article 972 (2018): 7884–7885.

[270] A. C. Komor , Y. B. Kim , M. S. Packer , J. A. Zuris , and D. R. Liu , “Programmable Editing of a Target Base in Genomic DNA without Double‐Stranded DNA Cleavage,” Nature 533 (2016): 420–424. 27096365 10.1038/nature17946 PMC4873371

[271] B. Y. Mok , M. H. de Moraes , J. Zeng , et al., “A Bacterial Cytidine Deaminase Toxin Enables CRISPR‐Free Mitochondrial Base Editing,” Nature 583 (2020): 631–637. 32641830 10.1038/s41586-020-2477-4 PMC7381381

[272] Z. Yang , X. Zeng , Y. Zhao , and R. Chen , “AlphaFold2 and Its Applications in the Fields of Biology and Medicine,” Signal Transduction and Targeted Therapy 8 (2023): 115. 36918529 10.1038/s41392-023-01381-z PMC10011802

[273] J. Huang , Q. Lin , H. Fei , et al., “Discovery of Deaminase Functions by Structure‐Based Protein Clustering,” Cell 186 (2023): 3182–3195. 37379837 10.1016/j.cell.2023.05.041

[274] T. Yuan , N. Yan , T. Fei , et al., “Optimization of C‐to‐G Base Editors with Sequence Context Preference Predictable by Machine Learning Methods,” Nature Communications 12 (2021): 4902. 10.1038/s41467-021-25217-y PMC8361092 34385461

[275] K. F. Marquart , A. Allam , S. Janjuha , et al., “Predicting Base Editing Outcomes with an Attention‐Based Deep Learning Algorithm Trained on High‐Throughput Target Library Screens,” Nature Communications 12 (2021): 5114. 10.1038/s41467-021-25375-z PMC8387386 34433819

[276] H. K. Kim , S. Min , M. Song , et al., “Deep Learning Improves Prediction of CRISPR–Cpf1 Guide RNA Activity,” Nature Biotechnology 36 (2018): 239–241. 10.1038/nbt.4061 29431740

[277] Q. Chen , G. Chuai , H. Zhang , et al., “Genome‐Wide CRISPR off‐Target Prediction and Optimization Using RNA‐DNA Interaction Fingerprints,” Nature Communications 14 (2023): 7521. 10.1038/s41467-023-42695-4 PMC10657421 37980345

[278] P. Patra , B. Disha , P. Kundu , M. Das , and A. Ghosh , “Recent Advances in Machine Learning Applications in Metabolic Engineering,” Biotechnology Advances 62 (2022): 108069. 36442697 10.1016/j.biotechadv.2022.108069

[279] Y. Wang , H. Wang , L. Wei , S. Li , L. Liu , and X. Wang , “Synthetic Promoter Design in Escherichia coli Based on a Deep Generative Network,” Nucleic Acids Research 48 (2020): 6403–6412. 32424410 10.1093/nar/gkaa325 PMC7337522

[280] H. Deng , H. Yu , Y. Deng , et al., “Pathway Evolution through a Bottlenecking‐Debottlenecking Strategy and Machine Learning‐Aided Flux Balancing,” Advanced Science 11 (2024): e2306935. 38321783 10.1002/advs.202306935 PMC11005738

[281] Y. Xia , X. Du , B. Liu , S. Guo , and Y.‐X. Huo , “Species‐Specific Design of Artificial Promoters by Transfer‐Learning Based Generative Deep‐Learning Model,” Nucleic Acids Research 52 (2024): 6145–6157. 38783063 10.1093/nar/gkae429 PMC11194083

[282] M. HamediRad , R. Chao , S. Weisberg , J. Lian , S. Sinha , and H. Zhao , “Towards a Fully Automated Algorithm Driven Platform for Biosystems Design,” Nature Communications 10 (2019): 5150. 10.1038/s41467-019-13189-z PMC6853954 31723141

[283] P. Kumar , P. A. Adamczyk , X. Zhang , et al., “Active and Machine Learning‐Based Approaches to Rapidly Enhance Microbial Chemical Production,” Metabolic Engineering 67 (2021): 216–226. 34229079 10.1016/j.ymben.2021.06.009

[284] C. J. Vavricka , S. Takahashi , N. Watanabe , et al., “Machine Learning Discovery of Missing Links That Mediate Alternative Branches to Plant Alkaloids,” Nature Communications 13 (2022): 1405. 10.1038/s41467-022-28883-8 PMC8927377 35296652

[285] S. P. Foy , K. Jacoby , D. A. Bota , et al., “Non‐Viral Precision T Cell Receptor Replacement for Personalized Cell Therapy,” Nature 615 (2023): 687–696. 36356599 10.1038/s41586-022-05531-1 PMC9768791

[286] J. Z. Williams , G. M. Allen , D. Shah , et al., “Precise T Cell Recognition Programs Designed by Transcriptionally Linking Multiple Receptors,” Science 370 (2020): 1099–1104. 33243890 10.1126/science.abc6270 PMC8054651

[287] C. Zhang , H. Liu , X. Li , F. Xu , and Z. Li , “Modularized Synthetic Biology Enabled Intelligent Biosensors,” Trends in Biotechnology 41 (2023): 1055–1065. 36967259 10.1016/j.tibtech.2023.03.005

[288] D. C. Fajgenbaum and C. H. June , “Cytokine Storm,” New England Journal of Medicine 383 (2020): 2255–2273. 33264547 10.1056/NEJMra2026131 PMC7727315

[289] X. Li , N. Gong , F. Tian , S. Zhang , et al., “Suppression of Cytokine Release Syndrome During CAR‐T‐Cell Therapy via a Subcutaneously Injected Interleukin‐6‐Adsorbing Hydrogel,” Nature Biomedical Engineering 7 (2023): 1129–1141. 10.1038/s41551-023-01084-4 37696984

[290] N. Gong , X. Han , L. Xue , et al., “In Situ PEGylation of CAR T Cells Alleviates Cytokine Release Syndrome and Neurotoxicity,” Nature Materials 22 (2023): 1571–1580. 37696939 10.1038/s41563-023-01646-6

[291] E. R. S. Cliff , A. H. Kelkar , D. A. Russler‐Germain , et al., “High Cost of Chimeric Antigen Receptor T‐Cells: Challenges and Solutions,” American Society of Clinical Oncology Educational Book 2023, e397912. 10.1200/EDBK_397912 37433102

[292] C. Roddie , M. O'Reilly , J. Dias Alves Pinto , K. Vispute , and M. Lowdell , “Manufacturing Chimeric Antigen Receptor T Cells: Issues and Challenges,” Cytotherapy 21 (2019): 327–340. 30685216 10.1016/j.jcyt.2018.11.009

[293] S. Pandit , P. Agarwalla , F. Song , A. Jansson , G. Dotti , and Y. Brudno , “Implantable CAR T Cell Factories Enhance Solid Tumor Treatment,” Biomaterials 308 (2024): 122580. 38640784 10.1016/j.biomaterials.2024.122580 PMC11125516

[294] S. Jo , S. Das , A. Williams , A. S. Chretien , et al., “Endowing Universal CAR T‐Cell with Immune‐Evasive Properties Using TALEN‐Gene Editing,” Nature Communications 13 (2022): 3453. 10.1038/s41467-022-30896-2 PMC9247096 35773273

[295] Y.‐R. Li , Y. Zhou , J. Yu , et al., “Generation of Allogeneic CAR‐NKT Cells from Hematopoietic Stem and Progenitor Cells using a Clinically Guided Culture Method,” Nature Biotechnology (2024). 10.1038/s41587-024-02226-y PMC11919731 38744947

[296] Y. Hu , Y. Zhou , M. Zhang , et al., “Genetically Modified CD7‐Targeting Allogeneic CAR‐T Cell Therapy with Enhanced Efficacy for Relapsed/Refractory CD7‐Positive Hematological Malignancies: A Phase I Clinical Study,” Cell Research 32 (2022): 995–1007. 36151216 10.1038/s41422-022-00721-y PMC9652391

[297] S. Badrinath , M. O. Dellacherie , A. Li , et al., “A Vaccine Targeting Resistant Tumours by Dual T Cell plus NK Cell Attack,” Nature 606 (2022): 992–998. 35614223 10.1038/s41586-022-04772-4 PMC10253041

[298] M. J. Dickinson , P. Barba , U. Jäger , et al., “A Novel Autologous CAR‐T Therapy, YTB323, With Preserved T‐Cell Stemness Shows Enhanced CAR T‐Cell Efficacy in Preclinical and Early Clinical Development,” Cancer Discovery 13 (2023): 1982–1997. 37249512 10.1158/2159-8290.CD-22-1276 PMC10481129

[299] N. Francis , M. Braun , S. Neagle , et al., Molecular Therapy—Methods & Clinical Development 31 (2023): 101114. 37790245 10.1016/j.omtm.2023.101114 PMC10544074

[300] J. Bailey , “CRISPR‐Mediated Gene Editing: Scientific and Ethical Issues,” Trends in Biotechnology 37 (2019): 920–921. 31182244 10.1016/j.tibtech.2019.05.002

[301] N. Kofler , “Why Were Scientists Silent over Gene‐Edited Babies?,” Nature 566 (2019): 427. 30809071 10.1038/d41586-019-00662-4

